# Effect of bisphenol A on the neurological system: a review update

**DOI:** 10.1007/s00204-023-03614-0

**Published:** 2023-10-19

**Authors:** Henrique Eloi Costa, Elisa Cairrao

**Affiliations:** 1 CICS-UBI, Health Sciences Research Centre, University of Beira Interior, Av. Infante D. Henrique, 6200-506 Covilhã, Portugal; 2 FCS-UBI, Faculty of Health Sciences, University of Beira Interior, 6200-506 Covilhã, Portugal

**Keywords:** BPA, Endocrine disruptor, Neurologic disease, Stroke, Plasticizers, Neurodevelopmental disease

## Abstract

Bisphenol A (BPA) is an endocrine-disrupting chemical (EDC) and one of the most produced synthetic compounds worldwide. BPA can be found in epoxy resins and polycarbonate plastics, which are frequently used in food storage and baby bottles. However, BPA can bind mainly to estrogen receptors, interfering with various neurologic functions, its use is a topic of significant concern. Nonetheless, the neurotoxicity of BPA has not been fully understood despite numerous investigations on its disruptive effects. Therefore, this review aims to highlight the most recent studies on the implications of BPA on the neurologic system. Our findings suggest that BPA exposure impairs various structural and molecular brain changes, promoting oxidative stress, changing expression levels of several crucial genes and proteins, destructive effects on neurotransmitters, excitotoxicity and neuroinflammation, damaged blood–brain barrier function, neuronal damage, apoptosis effects, disruption of intracellular Ca^2+^ homeostasis, increase in reactive oxygen species, promoted apoptosis and intracellular lactate dehydrogenase release, a decrease of axon length, microglial DNA damage, astrogliosis, and significantly reduced myelination. Moreover, BPA exposure increases the risk of developing neurologic diseases, including neurovascular (e.g. stroke) and neurodegenerative (e.g. Alzheimer’s and Parkinson’s) diseases. Furthermore, epidemiological studies showed that the adverse effects of BPA on neurodevelopment in children contributed to the emergence of serious neurological diseases like attention-deficit/hyperactivity disorder (ADHD), autism spectrum disorder (ASD), depression, emotional problems, anxiety, and cognitive disorders. In summary, BPA exposure compromises human health, promoting the development and progression of neurologic disorders. More research is required to fully understand how BPA-induced neurotoxicity affects human health.

## Introduction

According to the World Health Organization, stroke, or brain attack, is the most common neurovascular disease, the second most prevalent cause of death, and the third leading cause of long-term disability in the world. Unfortunately, the percentage of stroke victims is expected to increase in the next decade, affecting younger and younger patients (Sarikaya et al. 2015). Most strokes are Ischemic Stroke, approximately 70%, are caused by occlusion of the major cerebral artery, usually, the middle cerebral artery, leaving Haemorrhagic stroke, affecting about 30%, which forms lesions in the brain substance or subarachnoid space, among other rarer types in very small numbers (Iadecola et al. 2020).

Neurovascular diseases are a type of neurological disease and are of extreme concern to humans, as they can be fatal or cause permanent disability. Neurovascular diseases affect the cerebral vascular system and the spinal cord. These diseases can be caused by deformations in the endothelium layer, smooth muscle layer of blood vessels, and other molecular bases of pathogenesis (Sam et al. 2015). Apart from strokes, the most common neurovascular diseases are aneurysms, arteriovenous malformations, intracranial dural arteriovenous shunts, and spinal cord arteriovenous shunts, nevertheless, there are many other rarer neurovascular diseases (Song et al. 2021; van den Berg 2003). Most neurological diseases can arise from exposure to environmental chemicals or genetic predisposition (Yirun et al. 2021). Thus, recent studies highlight the role of vascular dysfunction in several neurodegenerative diseases, like Alzheimer’s and Parkinson’s, because the nervous and vascular systems are functionally interdependent and have close anatomical apposition as well as similar molecular pathways (Ahmad et al. 2020). Moreover, neurodevelopmental diseases, another type of neurologic disease, significantly impact children. The neurodevelopmental disorders more predominant are attention-deficit/hyperactivity disorder (ADHD), autism spectrum disorder (ASD), intellectual disability, and learning and communication disorders (Hansen et al. 2021; Thapar et al. 2017). These disorders significantly influence the lives of those who are affected and are linked to decreased daily functioning, academic performance, and life quality (Escobar et al. 2005).

Endocrine disruptors (EDCs), are synthetic or natural chemicals, usually environmental pollutants, that affect the endocrine system when exposed to humans from multiple sources, like atmosphere, water and effluents, food, drinking water, and dust, predominantly through breathing and food, acting to block endogenous hormones, and therefore can disrupt normal hormonal homeostasis, reproduction, and behaviour. EDCs have been severely used for various functions in recent decades, which facilitates their exposure to animals and humans (Klenke et al. 2016; Michałowicz 2014).

**Fig. 1:**
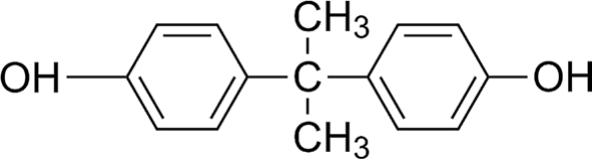
BPA chemical structure, drawn in ChemDraw®®

Of all the endocrine disruptors, bisphenol A (BPA) is a xenoestrogen that is one of the most produced synthetic compounds worldwide, with annual production exceeding 3.8 million tons and a release to the atmosphere of 100 tons in the same period (Michałowicz 2014; Vandenberg et al. 2010). BPA is mostly used as a monomer in the production of polycarbonate plastics and epoxy resins (Hoekstra and Simoneau 2013; Michałowicz 2014; Vandenberg et al. 2010). It is designated by IUPAC as 4,4′-ispropylidenediphenol (see Fig. 1), being a ubiquitous carbon-based chemical with chemical formula C_15_H_16_O_2_, formed by two phenol groups, with excellent physical and chemical properties (Banerjee et al. 2022; Campanale et al. 2020). Historically, BPA was synthesized in 1891 by a Russian chemist named Alexander Dianin, but it was not until about forty years later that some estrogenic effects were discovered (Rubin 2011). Nevertheless, this EDC is still used today because of the mechanical characteristics, low moisture absorption, elasticity and thermal resistance it provides to plastics (Michałowicz 2014).

As for its physicochemical characteristics, BPA is a chemical compound with a 228.29 g/cm^3^ molecular mass. Visually, BPA is white, solid, and crystalline. It has a melting point of 156 °C, with a boiling point of 220 °C (at a pressure of 5 hPa). Additionally, its water-octanol coefficient expressed in logarithmic form is 3.32 (log *P* = 3.32), which shows good solubility capabilities in fats and low water solubility, with about 200 mg/dm^3^ at 25 °C. Since BPA is a phenol, which can be converted to ethers, esters and salts, it has hydroxyl residues directly linked to its aromatic ring, which designates good reactivity to this compound (Fonseca et al. 2022). BPA binds to estrogen receptors such as ERα, ERβ, ERγ, G-protein-coupled estrogen receptor (GPR30) and peroxisome proliferator-activated receptor gamma (PPAR-γ) (Acconcia et al. 2015). The mechanisms of action are not yet fully understood, but several studies seem to show that BPA can induce insulin resistance, adipogenesis, pancreatic-β-cell dysfunction, inflammation, and oxidative stress (Banerjee et al. 2022).

Several studies have already reported numerous effects of BPA on various systems in the human body and in animals, as daily exposure to BPA has proven to be a major public health concern given its ubiquity and endocrine-disrupting properties (mainly estrogenic). However, the effect of BPA on the brain system is still an understudied topic, but it has been recognized that environmental exposure to BPA may affect some brain functions, interfering with brain development and physiology after bypassing endogenous defence mechanisms and may require exogenous interventions and that it may also be related to the prevalence of stroke (Cai et al. 2020; Di Pietro et al. 2020; Santoro et al. 2019). BPA neurotoxicity occurs in the brain system by reducing synaptic plasticity, inhibiting neurogenesis, creating oxidative stress, and inducting autophagy and apoptosis (Santoro et al. 2019). As such, we aim to address the disruptive effects of BPA on the human brain system by reviewing the current literature based on experimental studies in humans and animals and epidemiological data, with a focus on the underlying molecular mechanisms.

## Approach to the review

In this review, were presented and summarized experimental studies that evaluated the neurological effects caused by BPA exposure in animal, and human models, in Topic 3 and 4, respectively. In vitro and in vivo studies were the subtopics for this research looking at the effects of BPA exposure in animal models, while epidemiological studies and human cell lines were used as human models. A literature review was performed based on articles available in PubMed databases. The search strategy was carried out using Boolean operators “AND”, “OR”, and “NOT” and a combination of terms relating to Bisphenol A (“bisphenol A”, “BPA”, “neurotoxic”, “endocrine disruptor compound”) with brain system (“middle cerebral artery”, “vascular”, “vascular smooth muscle”, “smooth muscle cells”, “smooth muscle”, “brain”, “neurodevelopmental”) and neurovascular diseases (“stroke”, “cerebral infarction”, “vascular neurology”, “brain attack”, “neurodevelopmental diseases”, “neurodevelopment disorders”).

These terms were added to, along with the citations from the publications that were found through the search.

The inclusion criteria were: (1) the article was original; (2) the study was performed on in vivo animal models, in vitro models, or using epidemiological data; (3) the concentrations of exposure to BPA were well defined; (4) the studies had a control group; (5) the study analyses the neurological outcomes associated with exposure to BPA; (6) the study may also assess the effects associated with exposure to others EDCs; (7) the study evaluated the effects of BPA substitutes on other organ systems and characteristics; (8) the article was written in English. On the other hand, the exclusion criteria were: (1) the article was not original (e.g. review, editorial, and commentary); (2) studies including previous diseases or susceptible conditions without a control group; (3) the concentrations of exposure to BPA was not well established; (4) the study took place in the enteric nervous system, midgut or peripheral nervous system; (5) articles were duplicates, unrelated, inaccessible, or not written in English.

These inclusion and exclusion criteria were applied when each retrieved article's title, abstract, and materials and techniques were analysed.

## Exposure to BPA

The production and use of BPA have progressively increased every year, and this EDC is used in more and more types of materials, also expanding the number of contamination pathways, both through the use and wear of the containers, and through the pollution caused when they are disposed of in the aquatic, marine, air, and soil ecosystems (Le et al. 2008; Xing et al. 2022; Zhang et al. 2022a). Its existence in nature is only associated with human production activities, i.e. anthropogenic (Xing et al. 2022).

BPA is mostly used as a monomer in the production of polycarbonate plastics and epoxy resins used in the manufacture of dental fissure sealants, water supply pipes, adhesives, water bottles, food containers, electronic equipment, children’s toys, medical equipment, thermal paper, and others (Michałowicz 2014; Vandenberg et al. 2007). The most common contact consumers have with BPA is through the reuse of polycarbonate bottles and kitchen utensils, such as containers for use in the preservation and preparation of beverages and food or baby bottles, but they can also be subjected to this EDC through atmospheric air when it is in the gas form (Hoekstra and Simoneau 2013; Xing et al. 2022). Exposure to BPA can occur, most usually, by oral exposure through the ingestion of contaminated food and beverages or by skin contact or inhalation, and later accumulates in biological tissues, with likely long-term negative effects (Chianese et al. 2018b; Nunez et al. 2001). BPA can be released from containers when residues of the monomer in the polymer move into food and beverages, usually when the container is heated together with its contents, which in turn hydrolyse the polymer, promoting the release of BPA, or by diffusion of residual BPA present in the polycarbonate after the container manufacturing process (Hoekstra and Simoneau 2013; Xing et al. 2022).

**Fig. 2:**
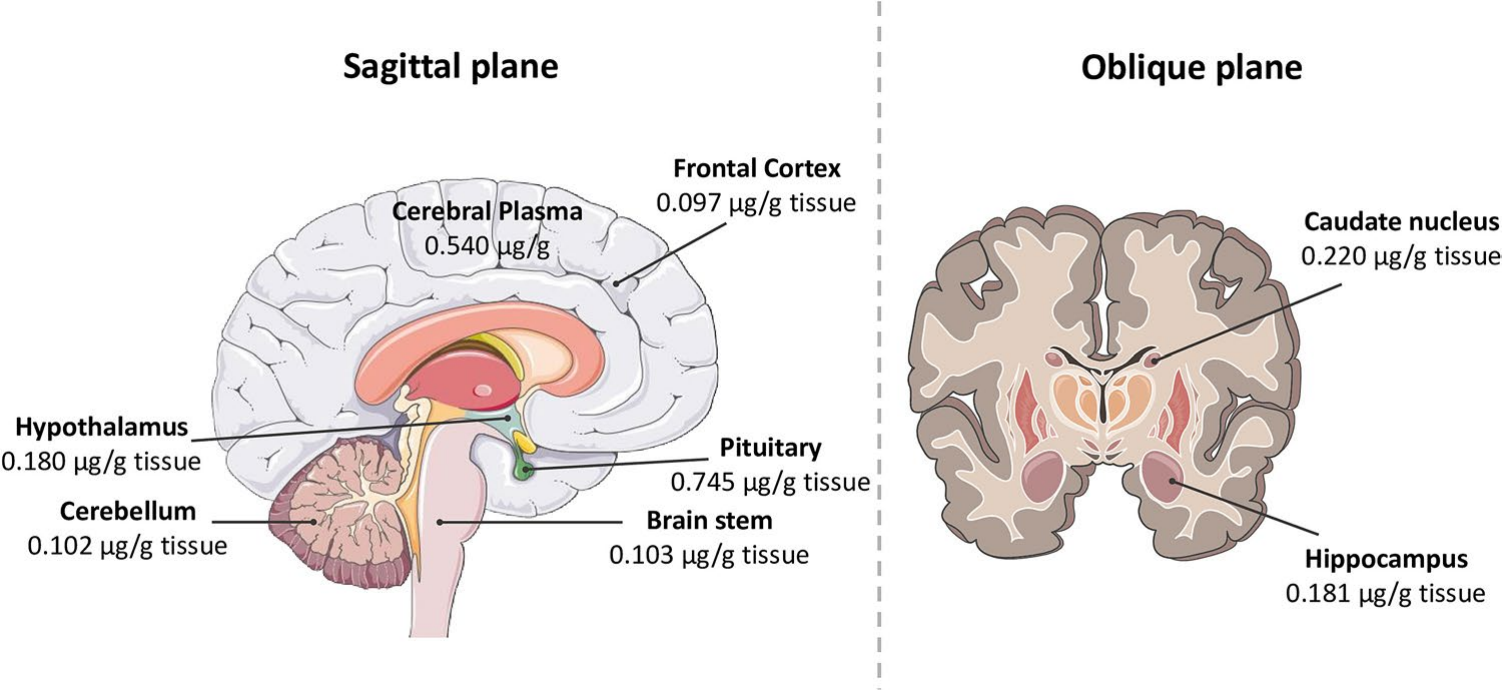
Accumulation of BPA in several brain regions 48 h after oral administration of 100 mg BPA/kg. The amount of BPA in each compartment is indicated as a μg BPA/g tissue. Data obtained by Kim et al. (2004). Figure created with PowerPoint version 2204 and using pictures from Servier Medical Art. Servier Medical Art by Servier, licensed under a Creative Commons Attribution 3.0 Unported License (https://creativecommons.org/licenses/by/3.0/)

In most cases, exposure to BPA is continuous, and its metabolites are detectable in about 90% of the world's population, but the degree of exposure to BPA may depend on socioeconomic factors, lifestyles, health status, exposure pathways and diet (Calafat et al. 2008; Ramadan et al. 2020). BPA is excreted mostly in urine as glucuronide/sulphate conjugates, occurs mostly within 24 h, has an estimated biological half-life of approximately 6 h, and is often used for biomonitoring studies (Braun 2017; Ramadan et al. 2020; Thayer et al. 2015). However, the small part that is not excreted, can be found in other biological fluids in lower concentration, such as maternal blood, maternal urine, amniotic liquid, placental tissue, umbilical cord blood, breast milk and human colostrum (Lorigo and Cairrao 2022; Santoro et al. 2019; Völkel et al. 2002). Regarding the concentrations of BPA that can be found at the brain level, Geens et al. (2012) detected BPA in the brain (0.91 ng/g) in samples taken in 2002 during the autopsy of eleven patients at the University Hospital of Antwerp (Geens et al. 2012). Furthermore, in a more detailed investigation of brain tissue, Kim et al. (2004), analysed the brains of Nulliparous Fischer 344 female rats administering an oral dose of 100 mg/kg BPA (see Fig. 2), and detected BPA accumulation after 48 h in plasma (0.540 μg/g), pituitary (0.745 μg/g tissue), hypothalamus (0.180 μg/g tissue), brain stem (0.103 μg/g tissue), cerebellum (0.102 μg/g tissue), frontal cortex (0.097 μg/g tissue), hippocampus (0.181 μg/g tissue) and caudate nucleus (0.220 μg/g tissue) (Kim et al. 2004).

Although there are several exposure pathways to BPA, most of the currently detected environmental concentrations of this compound for humans are below the current tolerable daily intake value of 0.2 ng per kilogram of body weight, established by the European Food Safety Authority (EFSA) in April 2023, a drastic reduction from the 4 μg/kg body weight/day established in 2015 (Kodila et al. 2023; Lambré et al. 2023; Silano et al. 2016). However, the tolerable daily intake rate is not completely reliable and safe, since the effects of BPA exposure are not always dose-dependent, as BPA has non-monotonic effects (Lorigo and Cairrao 2022), and BPA analogues have been developed to replace its use. However, these substitutes also appear not to be free of adverse effects, notably Bisphenol AF (BPAF) which shows higher neurotoxicity than BPA, but Bisphenol S (BPS) exhibits weaker neurotoxicity. Therefore, BPAF, BPF, and other analogues should be carefully considered as an alternative to BPA (Sam et al. 2015; Santoro et al. 2019).

The main harmful effects of BPA and its analogues are their acute and chronic effects on the brain, which is the organ responsible for controlling other organs, processing information, and supplying rapid and coordinated responses. An intricate neuronal network is formed during embryogenesis, but only in the postnatal period does the brain fully develop. Thus, the protection of neuronal cells and synaptic plasticity is crucial for healthy brain development. Conversely, neuronal damage, loss of synapses, and neurological disturbances are indicative of the development of neurological disorders and cognitive decline at the foetal level and later in the future generation (Chianese et al. 2018a; D'Angelo et al. 2019). Thus, in the prenatal phase, due to maternal exposure to BPA during the mother’s pregnancy, BPA crosses the blood–brain barrier and placenta (Balakrishnan et al. 2010) and is detected in the breast milk (Ye et al. 2006), and in the first years of a child's life, its neurodevelopment and normal behaviour can be compromised In this regard, exposure to EDCs during pregnancy is defined as “prenatal exposure” and can be subdivided into “maternal exposure” and “foetal exposure” (Lorigo and Cairrao 2022). In addition, common to the other EDCs, modulation of hormonal action by BPA occurs by specific cellular and molecular mechanisms, which may have additive, synergistic or negative biological effects. Consequently, the hormonal processes and signalling pathways that support the physiological changes that occur during pregnancy are affected, giving this EDC the possibility to increase maternal susceptibility and the risk of developing other complications in pregnancy, also affecting the foetus in the future (Lorigo and Cairrao 2022). Animal studies suggest that prenatal exposure to low doses of BPA may alter brain structure and function, and thus disrupt neurobehavioral development (Kundakovic and Champagne 2011; Kundakovic et al. 2013), whereas epidemiological studies have found the effect of maternal exposure to BPA at different times during pregnancy on neurobehavioral problems at children of different ages, especially in boys, often linking it to neurodevelopmental disorders (Evans et al. 2014; Harley et al. 2013; Jensen et al. 2019; Philippat et al. 2017).

Regarding the mode of action of this EDC, it can bind mainly to estrogen-activated receptors (ER), α and β (ERα and ERβ) and GPR30 receptors (Cimmino et al. 2020), and thanks to its chemical structure, BPA interacts as an antagonist or agonist, via a receptor-dependent signalling pathway. This EDC can also interact with other receptors, such as the androgen receptor (AR), estrogen-related receptor gamma (ERRγ), thyroid hormone receptor (THR), glucocorticoid receptor (GR) (Prasanth et al. 2010; Zhang et al. 2023), progesterone receptor (PR) (Huang et al. 2022a) and PPAR-γ (Acconcia et al. 2015; Di Pietro et al. 2020; Rubin 2011; Santoro et al. 2019). Still, the chemical structure of BPA may be an advantage, because BPA cannot adequately reach the boundaries of the hormone-binding site, only inducing a shift of α-helices forming the ligand-binding domain (LBD) (Acconcia et al. 2015). Overall, EDCs have three levels of key fragments: primary, secondary, and tertiary fragments. The secondary fragments are responsible for binding to receptors, which discriminate between active and inactive compounds, while the tertiary ones determine the type of activity, i.e., whether it is agonist, antagonist, or agonist–antagonist. This is defined by the interaction of EDCs with functional lobes, directly affecting the surface of AF-2, which handles the recruitment of coregulators. BPA holds primary fragments of oxygen-containing aromatics and secondary fragments (Bisphenol group). The activation of BPA, as an active compound, is given due to the coexistence of primary and secondary fragments. Through interactions of the secondary fragment of BPA, conformations stabilized in LBD, activation of the estrogen receptor and androgen receptor is achieved by forming hydrogen bonds with the amino acid R394 and via van der Waals interactions with the amino acid N705, respectively. Understanding the secondary fragments that form the LBD amino acid interaction network is critical to the activity of BPA. BPA ligand fragments interact with LBD causing changes in the conformation of the AF-2 surface, collecting two cofactors and identifying its tertiary fragment (agonist–antagonist activity) (Acconcia et al. 2015; Tan et al. 2020).

Similar to natural hormones, several experimental studies with BPA indicate a non-monotonic response, emphasizing the need for risk assessment with exposures from low to high dosages, given the typical U-shaped response also observed by other EDCs (Vandenberg 2014). However, this particularity may complicate the assessment of BPA toxicity risk, since this EDC can interact with hormone receptors on specific cell types and/or exhibit multiple biological endpoints with linear dose–response that cumulatively constitute a non-monotonic dose–response relationship (Cooper and Posnack 2022).

For these reasons, there has been increasing concern lately about the adverse effects of BPA exposure on human health. Accordingly, the European Food Safety Authority (EFSA) and the U.S. Food and Drug Administration (FDA) have imposed restrictions on the production and use of this EDC in infant feeding bottles and/or food plastic containers. These restrictions have also been adopted by other countries, such as Austria, Colombia, Canada, Denmark, France, and Belgium. Denmark and Sweden have also restricted the use of varnishes and coatings in food packaging for children (Boudalia and Malha 2016; Oliviero et al. 2022; Usman and Ahmad 2016).

That said, given the characteristics of BPA as an EDC and its continued exposure to humans, the endocrine-disrupting effects of BPA on the cerebral vascular system will be described in the following sections for animal models (Sect. Effects of BPA on Animal Models) and human models (Sect. Effects of BPA on Humans).

## Effects of BPA on animal models

Several studies have linked exposure to BPA to adverse effects on human health, mainly on neural, immune and metabolic systems, reproductive organs, and various cancers (Xing et al. 2022). However, recent evidence has also revealed a link between BPA exposure and neurovascular diseases, such as stroke, neurodegenerative diseases, like Alzheimer’s and Parkinson’s, and neurodevelopmental disorders, such as ASD and ADHD, for example (Cai et al. 2020).

In vitro and in vivo models are currently used to classify a substance as toxic. Therefore, in this section, we will address the neurotoxic effects of BPA in animals.

### In vitro studies

Exposure to BPA in animals has been reported in rodents, fish, and canines. In vitro studies are an indispensable tool for studying mechanistic pathways and can offer a faster and more flexible approach to human health effects. This type of study usually consists of exposing a certain type of cells to BPA. The in vitro studies analysed in this review have shown two major consequences caused by BPA exposure, regarding neuronal development and morphology, i.e., cell damage, and decreased cell viability, among others.

#### Neurodevelopmental effects

Concerning neuronal development, the first in vitro study analysed was conducted by Mathisen GH et al. The authors aimed to investigate whether prenatal exposure to BPA disrupted the neurodevelopment of the cerebellum. Thus, they exposed in vitro chicken embryos to BPA while still in the egg and prepared cerebellar granule cell cultures 24 h later. Experimentally, the authors saw that the level of Pax6 (a paired homeobox DNA binding protein) increased on day 6, and thus disrupted cerebellar development. Pax6 participates in the creation of neural circuits, neuronal migration, and brain patterning (Mathisen et al. 2013). In the same sense, Yin et al. analysed the effect of BPA exposure on mouse embryonic stem cells. These authors showed that BPA (1–10 μmol/L) affects the differentiation of germ layers during embryonic development, as well as the formation of neural ectoderm and neural progenitor cells. The authors concluded that studies in stem cells can be a powerful system for detecting the toxicity of environmental pollutants, such as BPA (Yin et al. 2015). In rat hippocampal neural stem cells (NSCs) Tiwari et al. studied the effect of BPA and concluded that exposure to 100 μM BPA significantly reduces the proliferation of NSCs, which reduces nestin, reelin, and Pax6 gene expression in the hippocampal region. Moreover, BPA also had inhibitory effects on neuronal differentiation, decreasing the number of newly born neurons and reducing long-term survival and further promoting increased phosphorylation of β-catenin, decreased levels of GSK-3β and nuclear translocation of β-catenin (Tiwari et al. 2016). Agarwal et al. in the same cells, found that chronic exposure to BPA (100 μM) impaired autophagy-mediated mitochondrial renewal, leading to increased oxidative stress, mitochondrial fragmentation, and apoptosis. Additionally, BPA also inhibited the proliferation and differentiation by decreasing the number of BrdU- and β-III tubulin-positive cells increasing levels of Drp-1 (dynamin-related protein 1) and increasing its mitochondrial translocation (which is associated with enhanced neurodegeneration and pathogenesis of neurodegenerative disorders), without difference in the hippocampus and in vitro levels for Fis-1, Mfn-1, Mfn-2, and Opa-1 (Agarwal et al. 2016). Furthermore, in the same cells from rat foetal, a comparison study of neurodevelopmental effects (cell proliferation, differentiation, and morphometric parameters) of BPA and Bisphenol F (BPF) was performed. The increase in cell proliferation and impact on differentiation rates of oligodendrocytes and neurons in a concentration-dependent manner was observed. Thus, the authors concluded that BPA and BPF induced similar changes in cell differentiation, proliferation and arborization (Gill and Kumara 2021). On the other hand, in gonadotropin-releasing hormone (GnRH) neurons, Klenke et al. investigated the effects of BPA on their activity using an explant model with a large number of primary GnRH neurons. These types of neurons are essential for reproduction, serving as an important link between the brain, pituitary, and gonads. These authors found that exposure to 50 μM of BPA significantly decreased calcium (Ca^2+^) activity and the GnRH neuronal activity, which occurred independently of estrogen receptors, GPER or ERRγ, via a non-canonical pathway, demonstrating a direct effect of BPA on GnRH neurons (Klenke et al. 2016). Conversely, Pang et al*.* investigated the transcriptional behaviour of long non-coding RNAs (lncRNAs) and mRNAs and discovered 151 lncRNAs and 794 mRNAs differentially expressed in the BPA-exposed group (2.5–200 μM). The differentially expressed mRNAs were involved mostly in basic metabolic processes as well as physiological and pathological conditions such as development, synaptic transmission, homeostasis, injury, and neuroinflammation responses (Pang et al. 2018). Besides, Cho et al*.* developed a method capable of effectively detecting neurotoxicity at low concentrations of BPA, in primary cultured mouse neurons. Thus, exposure to lower doses (50 µM and 100 µM) induced toxicity for developing neurons, especially impaired neuronal maturation in neural progenitor cells (Cho et al. 2018). In another in vitro study, the molecular mechanisms underlying the neurotoxicity of BPA and some analogues were investigated. The data show that 100 μM of BPA induced a 1.46-fold increase in O-GlcNAcase (OGA) protein level compared to the control. This protein performs a significant part in regulating neural activities at multiple levels, ranging from cellular processes to animal behaviours. Maintaining correct amounts of O-GlcNAcylation is necessary for embryonic development because a deficiency of OGA in mice causes mortality during embryogenesis or perinatal lethality, and therefore this OGA value is worrying. (Gu et al. 2019). Moreover, axon growth to assess the toxic effects of BPA (5 or 10 μM) on central nervous system (CNS) function and the role of protein disulfide isomerase (PDI) S-nitrosylation in BPA-induced neurotoxicity was studied. The authors concluded that BPA induced S-nitrosylation of PDI, while NG-monomethyl-l-arginine (L-NMMA), an inhibitor of nitric oxide synthases (NOS), exerted the opposite effects. However, BPA inhibited the growth of PC12 neurites, while L-NMMA reversed this inhibition (Kobayashi et al. 2020). On the other hand, in transgenic zebrafish cell lines, the neurotoxicity of 200 μg/L of BPA, BPF, BPAF and BPS was analysed, to complete the in vivo study, which will be mentioned in the next section. As a result, treatment with bisphenols led to significant reductions in green fluorescent protein (GFP) expression in the brain and spinal cord. GFP plays a key role in the development of zebrafish motor neurons. Similarly, the axon lengths of motoneurons at 36 h post-fertilization (hpf) were reduced by 40.4%, 25.2%, 33.0% and 46.2%, for BPA, BPS, BPF, and BPAF, respectively. (Gu et al. 2022). Conversely, in oligodendroglial cell lines (oli-neu), was examined how BPA affected their differentiation. BPA incubation decreased the amount of sulfatide and phosphatidylinositol in plasmalogen and changed the ratio of monounsaturated/polyunsaturated fatty acids in phospholipids, suggesting that BPA interferes with early oligodendrocyte differentiation’s lipid remodelling process (Naffaa et al. 2022). Furthermore, by treating PC12 cells, which is a cell line derived from a pheochromocytoma of the rat adrenal medulla, with BPA at 1 μM for 36 h, Bi et al. proved that exposure to this concentration of BPA impaired the neurite growth by decreasing primary and secondary branches. In addition, BPA exposure decreased the level of Ac-H3K9 by increasing the expression of HDAC2 (Bi et al. 2022).

#### Morphological effects

Regarding the morphology effects caused by BPA, 11 studies were conducted. Khan et al. showed that, in the C8-D1A mouse astrocyte cell line, BPA-induced cell damage (30 μM), such as cell loss, shorter cellular processes, and cell shrinkage, increased SYTOX-positive astrocytes and increased cell death by approximately 77% and expression of glial fibrillary acidic protein (GFAP) (Khan et al. 2018). Moreover, in mouse hippocampal HT-22 cell lines, the toxicity of BPA and its analogues showed that BPA, BPS and Bisphenol B (BPB) exposure increased reactive oxygen species (ROS) levels and apoptosis rates, damaged the cell membrane, and inhibited the proliferation (Pang et al. 2019). In another in vitro study, mouse neural stem cells were exposed to BPA and curcumin. The data showed a significantly decreased size and number of oligospheres in the BPA-treated group (100 μM) and a significant increase in the BPA + curcumin group. Additionally, a significantly decreased number of PDGFRα/PCNA + cells in the BPA-treated group was observed, while a significant increase in the BPA + curcumin group, suggesting that cell death was more prevalent in the presence of BPA (Tandon et al. 2020). In cultured Neuro-2a cells, which are a mouse cell line derived from mouse neuroblastoma tissue, Yin et al. assessed the effects of several concentrations of BPA (50, 100, 150, or 200 μM) on synaptic development and cytoskeleton. As a result, BPA altered synapse morphology by regulating synaptophysin (SYP) expression, causing a dose-dependent toxic effect. The expression of microtubule-associated protein 2 (MAP2), Tau decreased, disrupted microtubule stability and cell proliferation was also obtained, inducing cell death (Yin et al. 2020). Furthermore, in the same cells, there was another study, which investigated the effects and mechanisms of the BPA metabolite, 4-methyl-2,4-bis(4-hydroxyphenyl)pent-1-ene (MBP), on neuronal cell growth and function. MBP, which is generated in the mammalian liver after BPA exposure, is the main active metabolite of BPA, and the data showed that MBP exerts greater toxicity than BPA. MBP exposure significantly reduced the viability of Neuro-2a cells and induced apoptotic events in which MBP (5–15 μM) showed greater neuronal cytotoxicity than BPA (50–100 μM). BPA dramatically induced annexin V-FITC binding fluorescent intensity and sub-G1 hypodiploid cell population in a dose-dependent manner and resulted in marked expression of cleaved forms of caspase 3 and 7. Thus, these results allowed us to conclude that MBP exposure exerts neuronal cytotoxicity, via the interaction of the mitochondria-dependent and ER stress-triggered apoptotic pathway, which is regulated by ERK activation and Akt inactivation, leading to neuronal cell death (Huang et al. 2021). On the other hand, in cultured N2a neurons, that have similar properties to neuro-2a cells, were also exposed to BPA, with concentrations ranging up to 100 μM. The neurotoxicity of this EDC using viability and neuronal differentiation assays was performed, and the results showed that BPA decreased cell viability and axon growth (e.g., by down-regulating MAP2 and GAP43) and that it induced neurotoxicity caused by down-regulation of Bcl-2, initiating apoptosis, causing inhibition of autophagy flux, featured by nuclear translocation of apoptosis-inducing factor (AIF), light chain 3B (LC3B) aggregation, and p62 accumulation. Therefore, the authors confirmed that BPA induces neurotoxicity of neuro2a cells characterized by the (AIF)-dependent apoptosis and p62-related autophagy defects via positive regulation of heme oxygenase-1 (HO-1) and activation of AMP kinase (AMPK) resulting in neuronal degeneration (Lee et al. 2021). Moreover, in PC12 cells, the mechanism of neuronal apoptosis induced by various BPA concentrations (1, 10, 25, 50, 75, 100, 125, 150 μM) was investigated by the group of Zhang Y. In this study, BPA exposure reduced cell viability, altered cell morphology, and aggravated intracellular lactate dehydrogenase (LDH) release, intracellular Ca^2+^ concentration, ROS levels, apoptosis, and reduced mitochondrial transmembrane potential (ΔΨm). Furthermore, the authors linked BPA-induced apoptosis to Nur77-mediated inhibition of the NF-κb/Bcl-2 pathway (Zhang et al. 2021b). Nevertheless, on primary rat hippocampal neurons, Meng et al. studied the oxidative damage effects of different concentrations (1, 10, 100 nM and 1, 10, 100 μM) of BPA, BPS and BPB, and additionally evaluated the effects of epigallocatechin gallate (EGCG) (5 and 6 μM for males and females, respectively) added to BPA-exposed neurons. The results of this study showed that exposure to BPA, BPS and BPB can cause increased ROS levels and malondialdehyde (MDA) contents, but significantly reduced superoxide dismutase (SOD) activity and that they can also accelerate apoptosis and reduce cellular vitality of primary neurons. In addition, also concluded that males are more sensitive to bisphenols than females, and they hypothesized that this difference was due to the greater binding of this EDC to estrogen receptors (Meng et al. 2021). On the other hand, a study was tested in wild-type or histone deacetylase (HDAC2) silenced SN56 cholinergic cells, P75^NTR^ receptor or acetylcholinesterase (AChE) from the basal forebrain, to elucidate the mechanisms underlying the cognitive effects induced by BPA. Basal forebrain cholinergic neurons, regulate cognitive function and innervate the cortex and hippocampus, and their loss can cause cognitive disorders. Experimentally, BPA was administered, at concentrations between 0.001 μM and 100 μM, with or without recombinant nerve growth factor (NGF) and acetylcholine (ACh) for one and fourteen days. As a result, BPA induced disruption of cholinergic neurotransmission by reducing acetylcholine transferase (ChAT) activity causing decreased ACh and cell death, mediated partially by overexpression of AChE-S and dysfunction of NGF/TrkA/P75NTR signalling, through HDAC2 overexpression, independently of disruption of cholinergic neurotransmission (Moyano et al. 2021). Nevertheless, in mouse BV2 microglial cells, the research group of Sillapachaiyaporn C investigated the anti-neuroinflammatory effects of *Auricularia polytricha* against BPA-induced inflammation (0.01–100 µM). After exposure to the highest BPA concentration the levels of TNF-α, IL-1β and IL-6 mRNA expression were increased and a significantly decreased viability of BV2 cells after exposure for 24 and 48 h were also observed. There was still induction of inflammation, positively regulating the secretion of pro-inflammatory mediators through nuclear factor kappa B (NF-kB) signalling. These results suggest that BPA increased the secretion of pro-inflammatory cytokines in microglial cells. In addition, they used mouse hippocampal cells HT-22 to conclude that were damaged due to exposure to the same concentrations of BPA (Sillapachaiyaporn et al. 2022). Conversely, in a different study, on cortical neurons of embryonic mice, the effect of BPA exposure (0.01, 0.1, 1, 10, 100 and 1000 μM) was analysed, reporting changes in the morphology and function of synapses in the cerebral cortex. Cortical pyramidal neurons showed reduced size and number of dendrites and spines and the density of excitatory synapses decreased. Furthermore, BPA exposure impairs neurite growth and branching in cortical neurons and can disrupt normal synaptic transmission and cognitive behaviour. On the other hand, RGS4 and its downstream BDNF/NTRK2 pathway have been shown to mediate the effect of BPA on synaptic and neurological function (Hyun et al. 2022).

In summary, from this analysis, it seems clear that BPA exposure induces various brain changes at the functional level of the cortex and cellular and synaptic morphological modifications, as well as decreased cell viability and neuronal degeneration or defective development (Table 1). Moreover, it was observed that BPA promotes oxidative stress, originating from autophagy-mediated mitochondrial renewal, and originates alterations in the expression levels of several proteins, such as TNF-α, IL-1β, IL-6, nestin, reelin, SYP, MAP2, Tau, and HDAC2, of genes such as PAX-6, and lncRNAs. Thus, even while greatly remains unknown regarding the molecular effects of BPA on the brain system, it appears obvious that BPA exposure is linked to an increase in neurological pathologies.

**Table 1:** Summaries of the disruptive effects of BPA in animal in vitro studies

Topic	Animals/organs/cells	Drugs	Concentration	Results	References
Neurodevelopment	Chicken embryos	BPA	∼0.23 mg/kg	Increased Pax6 level in day 6, and thus disrupts cerebellar development	Mathisen et al. (2013)
	Mouse embryonic stem cells	BPA	1–10 μmol/L	Affected differentiation of germ layers during embryonic development and neuroectoderm Impaired embryonic development-based neural differentiation	Yin et al. (2015)
	NSC	BPA Curcumin	BPA (100 μM) and curcumin (0.5 μM)	Reduced NSC proliferation Inhibitory effects on neuronal differentiation Reduced expression of nestin, reelin, and Pax-6 gene in the hippocampus Decreased number of newly born neurons and their reduced long-term survival Increased β-catenin phosphorylation Decreased GSK-3β levels	Tiwari et al. (2016)
	NIH Swiss mice embryos	BPA	0.5 μM and 50 μM	Direct effect on GnRH neurons Decreased GnRH calcium activity in higher concentration BPA-mediated inhibition of GnRH neuronal activity occurred independent of GPER, or ERRγ, via a noncanonical pathway	Klenke et al. (2016)
	NSC	BPA	100 μM	Increased mitochondrial fission, oxidative stress, and apoptosis Inhibited proliferation and differentiation of hippocampus-derived NSCs Reduced number of BrdU- and β-III tubulin-positive cells Increased levels of Drp-1 Increased mitochondrial translocation of Drp-1	Agarwal et al. (2016)
	PC12	BPA	2.5, 5, 20, 40, 50, 80, 100 and 200 μM	Differentially expressed 151 lncRNAs and 794 mRNAs Aberrantly expressed lncRNAs	Pang et al. (2018)
	Primary neuron cultures of Sprague–Dawley embryonic rat brains	BPA	50, 100 or 200 μM	Toxicity at the highest concentration Affected neuronal maturation in neural progenitor cells at 100 µM of BPA Low concentrations toxic to developing neurons	Cho et al. (2018)
	PC12 cells	TBBPA, TCBPA, BPAF, BPB, BPA, BPF and BPS	0–100 μM	Increased OGA protein level by 1.46 folds, in higher concentration	Gu et al. (2019)
	Rat pheochromocytoma cell line (PC12)	BPA Rotenone	BPA (5 or 10 μM) Rotenone (100 or 200 nM)	Induced S-nitrosylation of PDI, with opposite effects in L-NMMA Inhibited neurite outgrowth of PC12, with L-NMMA reversed inhibition	Kobayashi et al. (2020)
	Rat foetal NSCs	BPA BPF	0.05 μM to 100 μM	Increased cell proliferation Impacted differentiation rates of oligodendrocytes and neurons Decreased maturation of oligodendrocytes and neurons Increased immature oligodendrocytes and neurons Increased astrocyte differentiation and morphological changes	Gill and Kumara 2021)
	Transgenic zebrafish cell lines	BPA BPF BPAF BPS	200 μg/L	Reduced GFP expression in the brain and spinal cord Axon lengths of motoneurons at 36 hpf were reduced by 40.4%	Gu et al. (2022)
	Oli-neu cells	BPA	0.01, 0.1, 1, 10, 20, 50, and 100 μM	Decreased amount of sulfatide and phosphatidylinositol in plasmalogen Changed ratio of monounsaturated/polyunsaturated fatty acids in phospholipids	Naffaa et al. (2022)
	Pheochromocytoma (PC12) cells	BPA	1 μM for 36 h	Impaired neurite outgrowth of PC12 cells Decreased level of Ac-H3K9 by upregulating expression of HDAC2 in PC12 cells	Bi et al. (2022)
Morphological changes	Mouse astrocyte type I cells (C8-D1A)	BPA α-Lipoic acid	30 μM BPA or α-Lipoic acid (100 μM) and BPA (30 μM) or α-Lipoic acid (100 μM)	Cell damage such as cell loss, shorter cell processes, and cell shrinkage Increased SYTOX-positive astrocytes Increased cell death by approximately 77% Increased expression of GFAP protein	Khan et al. (2018)
	HT-22 cell lines	BPA BPS BPB estradiol	1 nM, 10 nM, 100 nM, 1 μM, 10 μM, and 100 μM	Increased ROS levels Induced apoptosis Inhibition of cell proliferation Damaged cell membrane	Pang et al. (2019)
	Neural stem cells from embryonic day 12 rat embryos	BPA Curcumin	BPA (100 μM) or BPA (100 μM) and Curcumin (0.5 μM)	Decreased size and number of oligospheres in BPA treated group Increased size and number of oligospheres in BPA + curcumin group Decreased number of PDGFRα/PCNA + cells in BPA treated group Increased number of PDGFRα/PCNA + cells in BPA + curcumin group	Tandon et al. (2020)
	Neuro-2a cells	BPA DMSO	50, 100, 150, or 200 μM	Impaired morphology of synapse Decreased MAP2 and Tau expression Disrupted microtubule stability Dose-dependent toxic effect on Neuro-2a cells Inhibition of cell proliferation and cell death in high concentrations	Yin et al. (2020)
	PC12 cells	BPA	1, 10, 25, 50, 75, 100, 125, 150 μM	Reduced cell viability Altered cell morphology Aggravated intracellular LDH release, intracellular Ca2 + concentration, ROS levels, apoptosis Reduced mitochondrial transmembrane potential	Zhang et al. (2021b)
	Primary hippocampal neurons of Sprague–Dawley rats	BPA BPS BPB EGCG	1, 10, 100 nM and 1, 10, 100 μM	Accelerated apoptosis Reduced cell vitality Increased ROS levels and MDA contents Reduced SOD activity Males are more sensitive to BPs than females	Meng et al. (2021)
	Murine neuroblastoma cell line Neuro-2a	MBP BPA	BPA (0–300 μM) or MBP (5–15 μM)	MBP reduced Neuro-2a cell viability and induced apoptotic events MBP exhibited greater neuronal cytotoxicity than BPA BPA induced annexin V-FITC binding fluorescent intensity and sub-G1 hypodiploid cell population BPA resulted in marked expression of cleaved forms of caspase-3 and -7	Huang et al. (2021)
	Wild type or histone deacetylase (HDAC2), P75NTR or acetylcholinesterase (AChE) silenced SN56 cholinergic cells	BPA	0.001 μM–100 μM	Induced disruption of cholinergic neurotransmission Induced cell death Decreased ACh levels Disrupted NGF/TrkA/P75NTR signalling	Moyano et al. (2021)
	N2a neurons	BPA	0 to 100 µM	Decreased cell viability and axon outgrowth Induced neurotoxicity by down-regulating Bcl-2, initiating apoptosis and autophagy flux inhibition Induced neuro2a cells neurotoxicity	Lee et al. (2021)
	Mouse microglial cells (BV2 cell line) Mouse hippocampal cells (HT-22 cell line)	BPA	0.01–100 µM	Decreased BV2 cell viability in higher concentration Increased levels of TNF-α, IL-1β and IL-6 mRNA expression Induced inflammation in BV2 microglial cells Caused HT-22 hippocampal cell damage	Sillapachaiyaporn et al. (2022)
	Cortical neurons of embryonic mice	BPA	0.01, 0.1, 1, 10, 100 and 1000 μM	Altered synapse morphology and function in the cerebral cortex Reduced size of cortical pyramidal neurons and number of dendrites and spines Decreased density of excitatory synapses Impaired neurite outgrowth and branching in cortical neurons Disrupted normal synaptic transmission and cognitive behaviour Effect of BPA mediate RGS4 and BDNF/NTRK2 pathway on synaptic and neurological function	Hyun et al. (2022)

### In vivo studies

Regarding in vivo studies, the number of works currently available and included in this review is higher than in vitro studies, and these studies were mainly conducted in mammals, but there are also some studies in worms, fish, bivalves, insects, and amphibians, on the effect of this EDC on the cerebral vascular system. In this type of study, exposure to BPA is achieved in live animals and then the effects are evaluated.

#### Effect on worms

##### Neurodevelopment effects

The effect of BPA on neuroregeneration and neurodevelopment was recently analysed in worms. The asexual *Schmidtea mediterranea* CIW4 worms were exposed to 1.998 mg/L of BPA. BPA had severe, non-estrogenic effects on neuroregeneration in planaria, causing a severe lack of connection between nerve cords and longitudinal distribution from the brain primordia to the periphery of the head (Morris et al. 2022).

#### Effects on fish models

##### Morphological changes

Concerning the effect of BPA in relation to morphology alterations, neurodevelopment, neurobehaviour, neuroinflammation and neurovascular problems, several studies have been performed in two different species of fish. The zebrafish is a model commonly used in in vivo studies, in which animals are exposed to EDCs and the neurological effects are analysed, nevertheless, it is also possible and common to test on other fish such as goldfish. Regarding the morphologic changes caused by the BPA exposure, was seen predominantly phenotypical malformations and neuronal pyknosis. Therefore, a study in zebrafish embryos, subjected to an exposure of 100 nM and 100 μM of BPA and BPA analogues, and observed EDC-induced phenotypic malformations at 100 μM exposure, without effects at 100 nM (Björnsdotter et al. 2017). PK et al. exposed zebrafish males and females to 17.52 μM of BPA, which induced an increase of oxidative stress and neuronal pyknosis in the periventricular grey zone region of the zebrafish brain, which significantly altered the usual scototaxis behaviour (preference for darkness). Moreover, lipid peroxidation and protein carbonylation were increased, altering the antioxidant status of the brain and significantly decreasing the level of glutathione, glutathione reductase, glutathione-*S*-transferase and superoxide dismutase (Sahoo et al. 2020). Furthermore, adult zebrafish were exposed to 4 mg/L BPA and taurine, to evaluate the neuroprotective efficacy of taurine against BPA-induced neurotoxicity. As a result, taurine significantly ameliorated the effects of BPA exposure when exposed simultaneously, which were increased oxidative stress and neuronal pyknosis (Pradhan et al. 2021).

##### Neurodevelopmental effects

Thus, regarding the effects on zebrafish neurodevelopment, changes in different brain regions and even CNS homeostasis have also been observed. Moreover, in adult zebrafish, was determined the bioaccumulation potential of BPA (0.78 and 1 μg/L) in the absence and presence of plastic nanoparticles (1 mg/L), and various neurotoxic changes in the head. The results revealed that BPA can accumulate in the viscera, gills, head and muscles, and the co-exposure of nano-sized plastic particles (NPPs) and BPA resulted in a significant 2.2-fold and 2.6-fold increase in BPA uptake in the head and viscera. About the biomarkers, BPA treatment presented down-regulation of the anti-synapsin 2α (Syn2α) protein, which takes part in synaptic transmission. After the co-exposure of BPA and NPPs, the biomarkers of myelin and tubulin protein/gene expression, DA content and mesencephalic astrocyte-derived neurotrophic factor mRNA expression were significantly increased. In this regard, this study showed that BPA and NPPs can inhibit AChE activity. However, AChE activity was no longer inhibited in the co-exposure treatment. Finally, the authors also found that both BPA and NPPs can positively regulate myelin basic protein/gene in the CNS (Chen et al. 2017). Also in adult zebrafish, Guo et al. exposed them to 2 and 20 μg/L of BPA alone or together with 100 μg/L of titanium dioxide nanoparticles (n-TiO2). As a result, thyroid hormones (3,5,3′-triiodothyronine, T3 and thyroxine, T4) decreased on co-exposure, while T4 concentration in adult plasma decreased significantly because of exposure to 20 μg/L of BPA (Guo et al. 2019). On the other hand, in zebrafish during the early post-embryonic stage, a study evaluated the long-term effects of different bisphenols, with emphasis on brain development. Similar to its analogues, BPA showed accelerated embryo hatching rate effects, and in multiple brain areas, elevated levels of AroB protein expression were shown (Coumailleau et al. 2020). Conversely, in zebrafish embryos, evaluating the bioaccumulation of BPA (1, 4 and 20 μg/L) and 100 μg/L of titanium dioxide nanoparticles (n-TiO2), Fu et al. concluded that the transcript level of the neurodevelopment marker genes α1-tubulin, basic protein and Syn2α decreased in an exposure concentration-dependent manner, with significant differences in the 4 and 20 μg/L BPA groups (Fu et al. 2020). Furthermore, in the same animal and stage, in conjunction with a transgenic zebrafish model, Gyimah et al. performed BPA or BPS treatment. As a result, was caused hyperactivity, and interference with the normal expression of developmentally related genes vegfa, wnt8a and mstn1 at developmental stages. The expression of neurodevelopmental-related, ngn1, elavl3, GFAP, α1-tubulin, myelin basic protein and gap43, were also significantly increased, except for the myelin basic protein and GFAP gene that decreased considerably with exposure to 0.03 and 0.3 μM BPA, respectively (Gyimah et al. 2021). In the same sense, another study with zebrafish embryos, analysed various concentrations of Estrone or BPA. These two compounds exerted phenotypic or transcriptomic responses of about 1 nM of BPA. Exposure to estrone and BPA separately up to 5 days after fertilization caused non-monotonic transcriptomic changes, and exposure to BPA 4 to 5 days after fertilization caused hypoactivity and expression of neurological genes. Regarding short-term exposure, 100 nM BPA affected locomotive behaviour and altered the expression of 872 estrogenic and non-estrogenic genes, while at 10,000 nM there were 546 genes, with 122 genes differentially expressed at both concentrations that are linked to neurological diseases, neurodevelopmental included (Wu et al. 2021).

##### Neurobehavioural effects

In relation to neurobehaviour alterations, Li et al. exposed low doses of BPA (50 to 500 ng/L) for 7 weeks to adult male zebrafish, reporting physiological abnormalities and disruption of courtship behaviour (Li et al. 2017a). In the same conditions, the same authors published another study, reporting the effects of disruption of natural colour preference patterns, relief of anxiety-like behaviour, and altered ability to adapt to a different environment (Li et al. 2017b). Furthermore, in zebrafish embryos and larvae, Fraser TWK et al*.* showed that 10 μM BPA induced hyperactivity, hypoactivity, or had no behavioural effects (Fraser et al. 2017). However, in embryos, the research group of Zhang performed a rapid behavioural profiling assay that was designed to characterize the neurodevelopmental effects of environmental substances, such as BPA, by quantitatively assessing multiple spontaneous movement characteristics. These authors concluded that the frequency of spontaneous movement, in exposure to BPA at 1 and 10 μM, decreased significantly, but movement intensities increased by 41% for exposure with 1 μM of BPA and 19% at 10 μM, and also that intervals of spontaneous movement increased at 1 μM and 10 μM for BPA (Zhang et al. 2021a). Furthermore, the group of Sahoo PK exposed zebrafish to BPA (0.25, 0.5, 1, 2, 4.8, 16, and 32 mg/L) to discover the neurodegenerative potential of BPA in inducing Parkinson's disease-like phenotypes in zebrafish. As a result, pyknotic and Hoechst-positive neurons in the telencephalon and diencephalon were dramatically enhanced, as a measure of pyknosis and chromatin condensation. The concentration of 32 mg/L of BPA negatively affected neurobehavioural response, antioxidant status, and neuron morphology. Therefore, it was concluded that chronic exposure to BPA may induce neuropathological manifestations leading to the development of motor dysfunction and Parkinson-like neurodegenerative phenotypes in zebrafish (Sahoo et al. 2021). Besides, Gu et al*.* exposed the same species to 500 μg/L of BPA, which consequently had negative effects on exercise behaviour, CAT and SOD activity, larval development, gene expression in larvae, and behavioural inhibition, and also prevented the expression of CNS proteins in transgenic models (Gu et al. 2021). The same group also studied the neurotoxicity of 200 μg/L of BPA, BPF, BPAF and BPS. All bisphenols caused significant effects on embryo development at 200 μg/L, significant decreases in GABA neurotransmitters, increased concentrations of glutamate and glutamine and modification of the levels of five dopaminergic neurotransmitters (Gu et al. 2022). Moreover, the neuropharmacological effects of silibinin and naringenin were also analysed in zebrafish, against neurotoxicity and oxidative stress caused by 17.52 μM of BPA. BPA exposure induced neurobehavioural changes, such as altered scototaxis behaviour and spending more time in the upper zone compared to control groups, suggesting that the duration spent in the upper zone of the tank increased (Thayumanavan et al. 2022). In the same sense, Yang et al.*,* in zebrafish embryos and larvae, investigated the protective effects of Cyanidin-3-O-glucoside (C3G) against BPA-induced neurodevelopment damage. The data show that C3G co-exposed with BPA had significantly attenuating BPA effects, BPA was shown to have effects on locomotor behaviour and caused aberrant changes in brain morphology in zebrafish larvae. BPA further induced the decline of GSH, SOD, GPx CAT, oxidative stress and cell apoptosis (Yang et al. 2023).

##### Neurovascular and neuroinflammatory issues

Additionally, the effects of BPA on neurovascular and neuroinflammatory issues were analysed in two articles. Different from other experimental studies in zebrafish, the research work of Wang Q et al*.* consisted of submitting goldfish to 50 μg/L of BPA to understand the impact of this EDC in the brain. The data show that BPA can disrupt dopaminergic processes and damage blood vessels and induce cerebral atherosclerosis (Wang et al. 2019c). Moreover, zebrafish embryo models were used by Haridevamuthu et al. to evaluate the neuroprotective effects of Biochanin A against BPA-induced neuroinflammation. The results show that 1 µm of BPA dramatically elevated NO and LDH levels and inflammation-related expression levels, while considerably decreasing SOD, CAT, GST, GSH, AChE and Glutathione-related enzyme activity in the head region were observed. Oxidative stress damage and cell death were still brought on by prenatal exposure. In addition, BPA considerably reduced the speed of the larvae’s swimming speed and severely impaired the zebrafish's locomotor activity (Haridevamuthu et al. 2022).

#### Effects in fly models

##### Neurobehavioural effects

Two in vivo studies examining BPA effects in flies were conducted, reporting neurobehaviour and neurodevelopmental effects. Nguyen U et al*.* examined the effects of BPA on neurodevelopment in two genetic strains of fruit flies (Drosophila melanogaster) and looked at the phenotypes of the neurons and behaviour. BPA (0.1 and 1 mM) promoted recurrent grooming behaviour in adults, reduced cutting behaviour in larvae, inhibited axon guidance in the mushroom body, and interfered with neural stem cell formation in the genetic strain w1118. Therefore, this data suggests that BPA's effects on neurodevelopment can vary greatly depending on genetic background (Nguyen et al. 2021).

##### Neurodevelopmental effects

Welch et al. investigated the effect of BPA exposure in the development of fruit flies which affects gene expression, brain function, and synapse development. BPA mostly reduced the expression of genes involved in neurodevelopment, including those involved in learning, memory, and synaptic development as well as orthologs of human genes connected to neurological and neuropsychiatric disorders. In summary, BPA impaired associative learning and significantly increased the number of axonal branches (Welch et al. 2022).

#### Effects in frogs, mussels, tunicates, and clams

##### Morphological changes

Regarding the morphological effects of BPA, studies were also found in frogs, mussels, tunicates, and clams. Different impacts were seen, particularly concerning morphology, DNA, neurodevelopment, cell differentiation, and the expression of genes encoding modulating enzymes and receptors. On embryos and larvae of the South American common frog (*Rhinella arenarum*), Wolkowicz et al. analysed the acute and chronic toxicity of BPA, by continuous and pulse exposure. Embryos were treated continuously during early larval stages, and by pulse exposures of BPA for 24 h at concentrations between 1.25 and 40 mg/L. As a result, several morphological changes during the early stages were described, also impairing the gill circulation stage in all exposed organisms after 3 h of treatment with 10 mg/L BPA (Wolkowicz et al. 2014). Conversely, the same authors used the same animals and exposed them to concentrations of BADGE (Bisphenol A diglycidyl ether) evaluating the adverse effects of this organic compound through standardized bioassays. BADGE is a chemical compound related to BPA but with different properties and structure. The results showed that BADGE was more toxic to embryos than to larvae at all exposure times, and the most significant sublethal effects in embryos were cell dissociation and developmental delay, while in larvae it was related to neurotoxicity, response to stimuli and narcotic effect (Hutler Wolkowicz et al. 2016).

##### Neurodevelopmental effects

Concerning the neurodevelopmental effects, the research group of Juhel G exposed green mussels (*Perna viridis*) to different concentrations of carbamazepine, BPA, and the herbicide atrazine, analysing the effects on AChE. The exposure concentrations used for BPA were divided into low, medium, and high concentrations. However, the authors showed that BPA did not alter AChE activity but induced DNA damage, with increasing chemical concentrations (Juhel et al. 2017). Therefore, Gomes IDL et al*.* examined how BPA affected larval brain development of the ascidian *Phallusia mammillata,* which is a solitary marine tunicate of the ascidian class. Experimentally BPA was median effective in 11.8 μM and lethal in 21 μM of concentrations, whereas micromolar doses of BPA impaired differentiation of the ascidian-pigmented cells. The authors concluded that BPA may affect ascidian otolith differentiation (Gomes et al. 2019). Furthermore, Tang et al. exposed invertebrate bivalve species of clam (*Tegillarca granosa*) to 10 or 100 ng/L of BPA alone or together with 1 mg/L of microplastics. Exposure to BPA and microplastics led to an increase in three major neurotransmitters (Gamma-aminobutyric acid (GABA), DA, and Ach), and a decrease in the expression of genes encoding modulating enzymes and receptors for these neurotransmitters. In addition, the authors highlighted that the toxic effects exerted by BPA were significantly higher compared to previous studies due to the co-presence of microplastics (Tang et al. 2020).

#### Effects on rodent models

##### Morphological changes

Most of the articles reviewed in this section were experimentally performed in rodents, a total of 61 articles, resulting in multiple outcomes. In these studies, the main complications observed were related to morphological changes, altered neurodevelopment and neurobehaviour, alterations in specific brain areas, neuroinflammation, neurodegeneration and stroke-related problems. Thus, regarding the morphological alterations, El-Missiry et al*.* examined the protective effect of melatonin on oxidative stress and apoptotic death receptor proteins in the brains of BPA-treated rats. The results showed that BPA exposure induced significant increases in oxidative stress, and decreased glutathione levels and superoxide dismutase activity in the brain. In addition, BPA also caused upregulation of p53 and CD95-Fas and activation of caspases 3 and 8, resulting in increased apoptosis of brain cells (El-Missiry et al. 2014). In young male mice, the group of Zhou investigated the neuron toxicities of low doses of BPA. The percentages of DNA, tail length and timing in brain cells increased with increasing concentrations of BPA exposure. The authors found that low-dose BPA exposure can lead to DNA damage in brain cells, while prolonged exposure to this compound can impair learning and memory capacity (Zhou et al. 2017). Moreover, Di Pietro et al., in conjunction with a human in vitro experiment, examined the effects of 100 μg/L of BPA in glial and microglia cells of rat offspring at postnatal day 17 from pregnant females, who received BPA soon after coupling and during lactation and weaning. A significantly increased percentage of p-H2AX positive cells within IBA1 positive microglial cells in the hippocampal dentate gyrus, DNA damage in immune cells at the peripheral and central levels, a significant increase in the number of GFAP cells and a decrease in the marked Erα expression were observed. In this sense, they concluded that BPA exposure during development can induce microglial DNA damage and astrogliosis (Di Pietro et al. 2020). Additionally, the research group of Goyal S exposed Wistar rats to 40 μg/kg of BPA and observed a significant reduction in the levels of mitochondrial biogenesis proteins (PGC1α and TFAM) and mitochondrial import protein (GFER). In this regard, the authors demonstrated that BPA induced mitochondrial damage in neurons, reduction in GFER, translocation of cytochrome c to the cytosol and increased apoptosis (Goyal et al. 2021). Furthermore, Ishtiaq et al*.* administered 100 μg/kg, 1 or 10 mg/kg BW of BPA in adult Sprague Dawley rats for 16 days. BPA increased oxidative stress and increased expression of p53 upregulated modulator of apoptosis (PUMA), p53 and Drp-1, resulting in apoptosis in the brain tissue of rats (Ishtiaq et al. 2021). Bi et al. studied transgenic male Thy1-Cre mice and female C57BL/6 mice and exposed them to 0.5 mg/kg/day of BPA on a postnatal day. As a result, BPA reduced the number of RV + neurons in CA3 and entorhinal cortex but not in the medial septum, decreased the percentage of CaMKIIRV + cells in CA3 hippocampus and entorhinal cortex, and the synaptic connection of upstream glutamatergic neurons and CA1 pyramidal cells was weakened. They concluded that BPA induced detrimental effects on the excitatory neural circuitry of CA3-CA1 and EC-CA1 in memory formation (Bi et al. 2021). Moreover, the same authors exposed male and female C57BL/6 mice to 1 mg/kg/day of BPA for 10 weeks and observed an aberrant induction of HDAC2 expression. Nevertheless, was concluded that repression of HDAC2 could markedly rescue the impairment of spinal density in the hippocampus and prevent cognitive impairment caused by BPA exposure (Bi et al. 2022). On the other hand, El Tabaa et al*.* exposed Specific-pathogen-free adult white male Wistar rats to 250 mg/kg/day of BPA alone or together with 10 mg/kg/day of epigallocatechin-3-gallate (EGCG). BPA induced apoptotic and necrotic effects in the CA3 neurons of the hippocampus and a significant decrease in hippocampal concentrations of DA, norepinephrine (NE), 5-HT and Ach, as well as in hippocampal AChE activity in rats pre-exposed with EGCG for 2 h before BPA. Abnormal pyramidal cells in the hippocampus and increased hippocampal Casp-3 activity were also demonstrated (El Tabaa et al. 2022). In another study, in male Wistar albino rats, Caglayan et al*.* evaluated the exposure to 250 mg/kg of BPA, which significantly increased the MDA, NF-κB and TNF-α levels, and activated the JAK1/STAT1 signalling pathway. It also led to ER stress in brain tissue, regulated the levels of ATF-6, PERK, IRE1, and GRP78, and increased the mRNA levels of Bax and caspase-3, but insignificantly reduced the levels of Bcl-2 (Caglayan et al. 2022).

##### Neurodevelopmental effects

Concerning the neurodevelopment impact caused by BPA exposure in rodents, 18 studies have been performed and reported effects in impaired motor, spatial learning, memory development and cognitive function. Viberg et al*.* postnatal exposed NMRI mice to BPA (0.32, 3.2 or 4.8 mg BPA/kg body weight), concluding that can alter adult spontaneous behaviour and cognitive function and affect the cholinergic system. These effects were dose-dependent, long-lasting and irreversible (Viberg et al. 2011). These authors, in the same conditions, realized another study, indicating that a single postnatal exposure to BPA on postnatal day 10, during the peak of the brain growth spurt, can alter adult levels of important proteins for normal brain development (CaMKII and SYP) in both hippocampus and cerebral cortex (Viberg and Lee 2012). Conversely, in the prefrontal cortex (PFC) of adult rats, Castro et al. studied the effects of exposure to BPA for 4 days at 50 µg/kg of body weight and analysed the enzymes 5α-Reductase (5α-R) and cytochrome P450 aromatase (P450arom). The authors saw an increased expression of P450arom and isoform of tryptophan hydroxylase (Tph2) in the PFC of males and females but decreased the expression of 5α-R1 (an isozyme important in protecting neurons from apoptosis induced by glucocorticoid excess) in females. In addition, they also analysed 84 genes involved in BPA neurotoxicity in the adult PFC and identified 17 genes as potential targets of BPA, related to synaptic plasticity and memory functions (Castro et al. 2013). In pregnant Institute of Cancer Research (ICR) mice, Kumamoto et al*.* exposed pregnant mice to 0.02 or 50 mg/kg of BPA on gestational days 6 and 15 and examined neurodevelopmental-related genes linked to the X chromosome (Fmr1, Gdi1, Nlgn3, Pak3, and Ophn1) and genes related to sexual differentiation (ERα, ERβ, and AR). BPA caused a reduction in Xist, Fmr1, Gdi1, Nlgn3, and Pak3, and an increase in Tsix in the 50 mg/kg exposed group, while in the 0.02 mg/kg group, only moderately reduced Xist, Gdi1, Nlgn3, and Pak3. ERα, ERβ, and AR expression were changed in both exposure groups, and anogenital distance and estradiol levels were reduced (Kumamoto and Oshio 2013). On the other hand, Mathisen et al. analysed two strains of mice offspring who consumed BPA-contaminated water before mating, during gestation and lactation. As a result, BPA increased the total amount of Pax6 in the cerebellum and increased the thickness of the outer layer of granules in 11-day-old rat pups. This data agrees with in vitro results, obtained in the same study, which concluded that BPA may have an impact on the granule neurons responsible for the proper development of the cerebellum (Mathisen et al. 2013). The group of Nagao T developed a method to evaluate the behaviour of newborn rats to assess the risk of neurotoxicity of environmental toxicants by analysing motor activities including crawling, turning, straightening, or tremors. The newborn rats were exposed to 2, 20, or 200 µg/kg/day of BPA between days 5 and 18 of gestation and a significant increase in tremors on postnatal day 1 was observed (Nagao et al. 2014). Moreover, Zhang et al*.* in postnatal Sprague–Dawley rats exposure to a greater range of concentrations, 0.5–5000 μg/kg bw/day of BPA, concluded that BPA deteriorated the learning and memory capacity of rats, impaired the dendrites and spines, and disrupted the neurotransmitters homeostasis in the hippocampus. It was also observed that the neurotoxic effects of BPA exposure were different in males and females, i.e., sex-dependent differences (Zhang et al. 2019). The same group continued the study and observed that maternal exposure impaired spatial learning and memory capacity, altered hippocampal neurotransmitter levels, decreased neuron quantities and dendritic spine density across generations, and led to variations in hippocampal neurotransmitter levels (Zhang et al. 2020).

Concerting prenatal exposition, Castro et al*.* analysed the effect of a low dose of BPA (10 μg/kg/day) in male rats and studied the PFC, concerning dopamine (DA) and serotonin (5-HT) related genes modulated by BPA at the juvenile stage. The authors reported effects on gene transcription in juvenile and adult male rats and a reduction in precortical DA receptor transcripts in juvenile and Tph2 rats. They also found that there was increased tyrosine hydroxylase transcription in adult rats and that permanent changes were produced in glial cell-derived neurotrophic factor (GDNF) transcript levels (Castro et al. 2015). In pregnant Wistar rats, different concentrations of BPA were analysed by Hass et al*.* and neurodevelopmental effects were observed, after 25 μg/kg bw/day exposition. Moreover, female offspring experienced impaired spatial learning, which suggested a masculinization of the brain (Hass et al. 2016). Furthermore, Kimura et al*.* studied spine density and dendritic growth in the hippocampal CA1 of aged mice and developing mice exposed in the prenatal period, in utero, to 40 or 400 μg/kg BPA. Reduced spine densities in hippocampal CA1, decreased basal dendrite length and branching number and disrupted hippocampal CA1 neuronal morphology during development were reported, suggesting that this disruption may persist into adulthood (Kimura et al. 2016). On the other hand, in NCTR Sprague–Dawley, Arambula et al*.* concluded the transcriptome of the neonate amygdala can be disrupted by prenatal BPA exposure, whereby foetal development may make the female amygdala more vulnerable. BPA exposure affected the signalling pathways for oxytocin, vasopressin, and estrogen, which are important for the structure and transmission of synapses in the developing brain (Arambula et al. 2018). In the same sense, in Swiss albino mice, Birla et al*.* evaluated the effects induced by BPA (50 μg/kg bw/day) on spatial learning and oxidative stress. It was seen that BPA increased MDA levels in the hippocampus, and subsequently increased lipid peroxidation levels, inducing oxidative stress. Thus, the authors concluded that BPA significantly impaired mice’s spatial memory and learning (Birla et al. 2019).

In another in vivo study, adult male Wistar rats were exposed to 40 μg/kg of BPA by Tandon et al*.* BPA exposure induced reduced proliferation of oligodendrocyte progenitor cells in the hippocampus, as well as decreased learning and memory capacity, increased apoptosis, caused a decline in oligodendrocyte differentiation, resulted in altered myelin-specific gene expression profiles and protein levels, significantly down-regulated expression of Notch pathway genes, significantly decreased axon number. In addition to these effects, occurred a significant decrease in the intensity of Olig2/myelin basic protein + and myelin basic protein/neurofilament + co-localization in the hippocampus region (Tandon et al. 2020). Also, in adult male albino rats, exposed to 50 mg/kg bw of BPA, El Morsy et al*.* analysed the effect of BPA on proteins involved in neurodevelopment. The data showed a significant decrease in hippocampal GSH level by 53%, along with a five-fold increase in hippocampal MDA level, a significant decrease in the expression of hippocampal proteins TrKB, MAPK, ERK1/2 and CREB by 75%, 83%, 82% and 70%, respectively, along with a significant decline in hippocampal brain BDNF level by 46%. Additionally, significantly reduced hippocampus AChE activity by 50% and caused a massive increase in the hippocampal pro-apoptotic gene Bax (fivefold), and accelerated hippocampus Caspase-3 activity (fourfold), accompanied by a significant decrease in the hippocampal antiapoptotic gene Bcl-2 (4.5-fold) (El Morsy and Ahmed 2020). Nevertheless, Mao et al*.* studied for the first time the effect of BPA in small RNAs and found the mRNAs and the pathways of microRNAs (miRNAs) in the rat placenta. Therefore, 43 small RNAs were differentially expressed, and target mRNAs closely matched those expressed by the brain and thymus, demonstrating their ability to control neurogenesis and related neurodevelopmental processes. Thus, the authors concluded that the placenta might influence foetal brain development via secreting miRNAs (Mao et al. 2021). In adult female Swiss albino rats, Elbakry et al*.* evaluated the enhancing effect of nattokinase against liver and brain damage induced by 100 mg/kg bw/day of BPA or 3 Gy/week of γ-IR. BPA caused increased levels of the misfolded proteins Aβ and p-tau, induced cognitive deficits and impaired learning memory in offspring mice, and decreased acetylcholine transferase activity (Elbakry et al. 2022). Conversely, also in the mouse hippocampus, Luo et al*.* investigated BPA's impact on the miRNA expression profile to learn more about the miRNA function in BPA-induced learning and memory impairment. As a result, BPA had negative effects on spatial learning and memory as well as alterations in the miRNA expression. In addition, 17 miRNAs significantly altered expression levels (Luo et al. 2023).

##### Specific brain area modifications

Regarding the effect of BPA exposure in rodents, some studies, have reported modifications in specific brain areas. Khadrawy et al*.* analysed the effect of exposure to two doses of BPA (10 and 25 mg/kg) in adult male albino rats on excitatory (of glutamate and aspartate) and inhibitory (of γ-aminobutyric acid, glycine, and taurine) amino acid neurotransmitter levels in the cortex and hippocampus. As a result, in the cortex, there was a significant increase in excitatory amino acids, lipid peroxidation and nitric oxide (NO), although a significant decrease in inhibitory amino acids and glutathione. In the hippocampus, there was a significant increase in excitatory and inhibitory amino acid neurotransmitters, such as lipid peroxidation and reduced glutathione. In addition, the authors investigated the effect of BPA on AChE activity, and it induced a significant increase in cortical and hippocampal AChE activity. Thus, the authors concluded that BPA induced a state of excitotoxicity and oxidative stress (Khadrawy et al. 2016). In the same sense, Tavakkoli et al*.* studied the changes of proteins in the cerebral cortex and exposed adult male Wistar rats to 0.5, 5 and 50 mg/kg of BPA. At 0.5 and 5 mg/kg, lipid peroxidation was significantly higher than in the control group and showed some pro-oxidant effects. There was an 80% increase in pyruvate kinase M2 (PKM2) levels, and PKM2 expression was also increased in some cancer cells, resulting in phosphoenolpyruvate accumulation. The authors further indicated that the expression of 10 proteins was altered, which has already been associated by other authors with neurological and psychosocial disorders, including neurodegenerative diseases, schizophrenia, depression, epilepsy, and some brain tumours (Tavakkoli et al. 2020). The group of Santoro A continued the study of Di Pietro et al*.* using the same animals and treatment with BPA (Di Pietro et al. 2020; Santoro et al. 2021). However, different results were obtained, reporting enlarged lateral cerebral ventricles in lactating and weaned BPA-exposed animals. BPA exposure also affected the expression of inflammatory cytokines, Sirt1, its natural antisense long non-coding RNA (Sirt1-AS LncRNA) and histone deacetylase 1 (HDAC1) (Santoro et al. 2021). Moreover, Essawy et al*.* investigated the effect of astragaloside IV or saponins extracted from *Astragalus spinosus* on DNA damage, which was induced by 125 mg/kg/day of BPA in the PFC, hippocampus, and striatal brain regions of developing male rats. On exposure to BPA, increased levels of NO and decreased levels of glutamate, glutamine, glutaminase and glutamine synthetase were seen, inducing marked DNA damage in the brain regions studied. BPA also markedly decreased BDNF concentration in the hippocampal and striatal regions, although insignificantly affected the PFC. Additionally, BPA exposure resulted in the upregulation of NR2A mRNA expression levels in the PFC and hippocampus and its downregulation in the striatum, while NR2B mRNA expression level was downregulated in PFC and hippocampus regions, but upregulated in the striatum brain region (Essawy et al. 2021).

Furthermore, the group of Yirun A exposed pregnant Sprague–Dawley rats, during pregnancy and lactation, to 50 mg/kg/day of BPA. In this study, BPA exposure led to apoptosis, histopathological changes in the hippocampus, altered GSH and MDA levels, and AchE activity. The results further showed that exposure to EDCs in early life stages caused significant changes in lipid peroxidation, total neurotransmitter levels, and activities of neurotransmitter-related enzymes (Yirun et al. 2021). In the same sense, Zheng et al*.* investigated the effects of BPA levels in a mouse model on the effects of maternal BPA exposure on children's brain development. Regarding the in vivo study, the authors exposed CD-1 mice to 2.25 μg/kg bw/day of BPA and concluded that provoked structural alterations in brain regions including the superior olivary complex and bed nucleus of stria terminalis with larger effect sizes (Zheng et al. 2022). Sevastre-Berghian et al*.* evaluated the effects of BPA with or without melatonin, in 2-month-old male Wistar rats. As a result, BPA exerted an inhibitory effect on general locomotion and was induced in the frontal lobe and the hippocampus activation of MAPK. The monocyte chemoattractant protein-1 (MCP1) expression was reduced and the expression of pNFkB was considerably enhanced. MCP1 is expressed by neurons, astrocytes, microglia, and capillary endothelium in the brain, and may alter the permeability of the blood–brain barrier and modulate various neuronal lesions (Yao and Tsirka 2014). As a result, in groups that had been exposed to BPA, there was cellular oedema, necrosis, and a polymorphic cellular inflammatory infiltration that increased the intercellular spaces (Sevastre-Berghian et al. 2022).

##### Neurobehavioural effects

Concerning the neurobehavioural effects in rodents, 10 articles were analysed. The first was performed in 2011 by Ishido et al*.* who examined the effects of BPA and two derivatives, on the behaviour of 5-day-old male Wistar rats, concluding that at 20 μg of BPA, there was an increase in hyperactivity after 4 to 5 weeks of exposure. They also reported that there was increased activity in the nocturnal phase by 1.3 times (Ishido et al. 2011). Moreover, Luo et al*.* exposed male mice to a 50 mg BPA/kg diet per day for 35 days, and observed an increased anxiety-like behaviour, although in the hippocampus, AChE activity was dramatically reduced (Luo et al. 2013). Furthermore, Kundakovic et al*.* observed the effects of long-term BPA prenatal exposure to 200 μg/kg per day on the behaviour of mice and concluded that there was an induction of long-lasting changes in DNA methylation in the transcriptionally relevant region of the derived neurotrophic factor (BDNF) gene in the hippocampus and blood (Kundakovic et al. 2015). On the other hand, in young and adult NCTR Sprague–Dawley rats, Rebuli et al*.* evaluated the impact of perinatal exposure to BPA (2.5, 25 or 2500 µg/kg body weight) on anxiety-related behaviours and exploratory activity. Several parameters on the behaviour were evaluated inside a 50 cm long and 10 cm wide apparatus. Differentially, two arms were placed inside 40 cm walls and two arms with a short (8 mm) overhang around the edge. No consistent effect was seen, in either sex, at either age compared to vehicle controls. However, in comparison with the exposure to ethinyl estradiol (0.5 µg/kg bw/day), which was used as the reference estrogen, significant differences were found for some parameters, such as time spent and distance walked on the apparatus, in different measurements compared to the control (Rebuli et al. 2015). The neuromodulatory effect of α-Lipoic acid against BPA neurotoxicity (10 mg/kg) in male C57BL/6 J mice was analysed by Khan et al. The data obtained showed that the activity of monoamine, the level of lipid peroxidation and the content of protein oxidation were significantly increased, while AChE, catalase (CAT), glutathione-*S*-transferase (GST) and glutathione (GSH) activities decreased. The combined treatment of BPA and α-Lipoic acid showed a significantly higher level of GSH (Khan et al. 2018). Furthermore, on rats, the group of Henriksen AD analysed the impact of BPA exposure, on the anterior hypothalamus, the basal nucleus of the stria terminalis, and hippocampal gene expression. 259 genes were overrepresented in categories related to mating, cell–cell signalling, behaviour, neurodevelopment, neurogenesis, synapse formation, cognition, learning behaviours, hormone activity, and signalling receptor activity, among other things, in the mice exposed to BPA (Henriksen et al. 2020). Besides, Repouskou et al*.* used a mixture of phthalates, pesticides, and BPA in rats and human epidemiological studies, which were used to better mimic real-life situations. This mixture was exposed to mice throughout gestation and increased active coping during swimming stress in both sexes, although just in male offspring, increased locomotion, decreased social interaction and altered the expression of all examined genes (Nr3c1, Crh, Fkbp5, Oxt, and Esr2) in the hypothalamus (Repouskou et al. 2020). In Wistar albino rats, Singha et al*.* exposed 5 mg/kg body weight in prenatal and postnatal periods to clarify how astrocyte shape and GABA transmission contribute to the cognitive impairments caused by BPA. BPA during and after pregnancy caused anxiety-like behaviour, decreased serum GABA levels and was observed reduced dendritic spine counts and astrocyte numbers (Singha et al. 2021). Following that, Gore et al*.* used a mixture called NeuroMix, that have bisphenols, phthalates, vinclozolin and perfluorinated, polybrominated and polychlorinated compounds, and exposed pregnant Sprague Dawley rats. Adult females were the only ones affected by NeuroMix's anxiety-like, social, and mate-preference behaviours, but stress had more impact on males than females. In summary, many relationships between NeuroMix and stress were discovered, particularly for brain gene expression and partner choice behaviour (Gore et al. 2022). On the other hand, the group of Hyun wanted to confirm the effect of exposure to BPA (50 µg/kg) for 2 months in Mice (C57BL/6N). In this case, BPA exposure resulted in high anxiety behaviour and disrupted memory processing, in agreement with the in vitro experiments (Hyun et al. 2022).

##### Stroke-related problems

Regarding the interaction of BADGE and its influence on cerebral ischemia, reperfusion injury, and ischemic stroke through a middle cerebral artery occlusion treatment (MCAo) or a middle cerebral artery occlusion and reperfusion (MCAO/R), three studies have been performed also in rodents. Therefore, the research work of Certo et al*.* evaluated the participation of retinoid X receptors (RXR) in the development of ischemic stroke injury. The experiments were performed in adult male mice, with 5 experimental groups, where in the last group, MCAo was performed for 30 min after administration of Bexarotene or 30 mg/kg of BADGE. The BADGE, which is a PPAR-y antagonist, was used to serve as an antagonist to improve histological outcomes and also because of the ability of bexarotene to reverse spleen atrophy induced by middle cerebral artery occlusion. Pharmacological blockade of PPAR-γ, with BADGE, significantly reversed the reduction in cerebral infarct volume (Certo et al. 2015). Subsequently, in male rats, Wang et al*.* induced the MCAO/R (2-h occlusion, followed by reperfusion for 48 h). The authors aimed to examine how ursolic acid acts as a neuroprotective agent to modulate the metalloprotease/anti-metalloprotease balance. They posteriorly administered ursolic acid intragastrically 0.5, 24 and 47 h after reperfusion, and BADGE intraperitoneally 1, 24.5 and 47.5 h after reperfusion. The exposure to BADGE was 30 mg/kg alone or together with 5, 10 or 20 mg/kg ursolic acid. A significant elimination of the ursolic acid-induced enhancement in PPAR-γ expression was shown. BADGE also provoked a partial and significant block of metalloproteinase-2 (MMP2) and MMP9 levels, although increased metallopeptidase inhibitor 1 (TIMP1) levels, were previously induced by ursolic acid. (Wang et al. 2016). Following that, Wu et al*.* investigated the alterations of the PPAR-γ-activated gamma receptor in focal cerebral ischemia–reperfusion injury. They experimentally performed the rat model of middle cerebral artery occlusion and reperfusion and exposed male Sprague–Dawley rats to 30 mg/kg BADGE, in one of the experimental groups. MCAO/R significantly increased neurological deficit scores, TNF-α, IL-1β and IL-6 levels (Wu et al. 2018).

##### Neuroinflammation effects

Concerning neuroinflammation effects after BPA rodent exposure, 4 studies were analysed. In hypertensive adult male Wistar rats, exposed to 100 mg/kg of BPA, Akintunde et al*.* observed increased motor coordination and antigen-presenting cells and CD43 T-killer cells, and also regulated pro-inflammatory mediators (Akintunde et al. 2020). On the other hand, Ni et al*.* exposed 8-week-old adult C57BL/6J mice to 0.05, 0.5, 5 and 50 mg/kg BPA. BPA exposure was found to induce cognitive impairment in males, but not in females, which may be caused by microbial gender differences and the role of estrogen receptors. BPA further increased neuroinflammation and damaged blood–brain barrier function, caused intestinal barrier dysfunction, altered microbiota composition, and also decreased levels of 5-HT and its metabolites in serum and hippocampus (Ni et al. 2021). Moreover, Abdou et al*.* exposed adult male Wister albino rats to 50 mg/kg bw/day of BPA. Exposure to BPA led to a significant increase in brain MDA and NO levels, associated with a significant reduction in GSH, total thiols, glutathione peroxidase (GPx), SOD and GST. Serum levels of total cholesterol, triglycerides, and low-density lipoprotein cholesterol (LDL) were increased, and an increase in specific AChE activity and DA and 5-HT levels occurred. The expression of inflammatory markers (tumour necrosis factor-α (TNF-α), cyclooxygenase-2 (COX-2), and tumour suppressor and pro-oxidant p53 protein (p53 genes) were also increased (Abdou et al. 2022). Furthermore, Lama et al*.* examined the impact of 50 μg/kg of BPA on anxiety-like behaviour in obese mice, and whether it is linked to neuroinflammation and immune activation. The data show that BPA deteriorated the anxiety-like behaviour caused by the high-fat diet (HFD) and the neuroinflammation brought on by HFD, which also increased IL-1, TNF-, and MCP1 in PFC. BPA generated monocyte recruitment and inflammation in the hypothalamus and amygdala. Nonetheless, the authors concluded that subchronic exposure to BPA increased neuroinflammation and immunological activation brought on by the HFD diet, constituting an extra risk factor for mood disorders that are solely connected to obesity (Lama et al. 2023).

##### Neurodegeneration effects

Regarding the neurodegeneration effects caused by BPA exposure in rodents, ten articles have been analysed. The research group of Agarwal et al*.* attempted to demonstrate that autophagy acts as a transient cellular protective response against BPA-induced neurotoxicity. These authors saw their effect during the early postnatal period of C57BL6 male mice and saw an increased expression and levels of genes/proteins involved in autophagy. BPA exposure further negatively regulated the rapamycin (mTOR) pathway promoting mitochondrial loss, bioenergetic deficits and increased mitochondrial translocation of PARKIN, which is a cytosolic E3 ubiquitin ligase. In the long term, decreased levels of Lamp-2 and positive regulation of p62 and cleaved caspase-3 were seen. Thus, the authors concluded that autophagy may protect against BPA-induced neurotoxicity by activating the AMPK and mTOR pathways (Agarwal et al. 2015). Moreover, in another in vivo study, the same authors concluded that chronic exposure to BPA causes mitochondrial dynamics, leading to neurodegeneration, and apoptosis in the rat brain hippocampus, in addition to autophagy. Experimentally, Wistar rats to 40 μg/kg body weight were exposed to BPA, which increased Drp-1 levels, associated with impaired autophagy, increased apoptosis, and increased the levels of Drp-1, which is a dynamin-related protein, in hippocampal tissue (Agarwal et al. 2016). On the other hand, Tiwari SK et al. exposed adult female Wistar rats to the same BPA concentration. The effects observed were a decrease of the antiapoptotic gene Bcl-2 and an upregulation of the gene Bax, thus a significant increase in neural degeneration (Tiwari et al. 2016). Furthermore, Li et al*.* examined the effects of 30-day exposure to 100 μg/kg/day of BPA on glucose transport and the IR/IRS/AKT/GSK3β axis in adult male mice to determine if there is an association between disruption of insulin signalling and neurotoxicity of this EDC. The authors demonstrated a decreased insulin sensitivity, decreased glucose transporter protein 1, 3 (GLUT1,3) levels, hyperactivation of the IR/AKT/GSK3β axis, and increased phosphorylated tau and β-APP. In conclusion, BPA exposure may be a potential risk factor for neurodegenerative diseases, as these results strongly indicate significant disruption of insulin signalling in the brain (Li et al. 2016). Nevertheless, Khan et al*.* examined whether exposure to 40 and 400 μg/kg body weight of BPA alters the expression of axial and myelin structural proteins in the cerebral cortex of C57BL/6J mice. BPA causes oxidative stress in the brain, memory loss, muscle coordination deficits, allodynia, degeneration of mature and immature oligodendrocytes, nestin, myelin basic protein, and also myelin-associated glycoprotein-1 (MAG-1). Subchronic exposure to BPA caused neuroinflammation through the downregulation of inflammatory cytokine mRNA and protein expression. In conclusion, the authors considered that long-term exposure to 40 μg/kg body weight of BPA may cause axonal degeneration and demyelination in oligodendrocytes and neurons, and may be involved in the development of neurological and neuropsychological disorders, including multiple sclerosis, neuromyelitis optics (Khan et al. 2019). In adult female Wistar albino rats, Abdel-Rafei et al*.* analysed the modulatory role of methylsulfonylmethane (MSM), in combating neurodegenerative disorder (Alzheimer's mimicking neurotoxicity), induced by co-exposure to BPA and gamma radiation (γ-IR). It was found that co-exposure of BPA and γ-IR induces hallmarks typical of neurodegenerative disorders, due to rather high oxidative stress, extensive neuroinflammation, elevated Alzheimer’s markers in the cortex and hippocampus, as well as up-regulation of microglial pro-inflammatory triggering receptor expressed on the myeloid cell-2/DNAX-activating protein of 12 kDa/spleen-tyrosine kinase pathway and its downstream targets (PLC-γ/DAG/p38-MAPK) in the hippocampus. In addition, neurodegenerative lesions were detected on histopathological examination of the cortex and hippocampus along with marked Aβ deposition in the hippocampus (Abdel-Rafei and Thabet 2020). Moreover, in male Sprague–Dawley rats, the research group of Kobayashi analysed the exposure effect of 100 mg/kg/day of BPA or 3 mg/kg/day of Rotenone on the cerebrovascular system. Thus, the data demonstrated that BPA and rotenone-induced S-nitrosylation of protein disulfide isomerase in brain microsomes, which can lead to Alzheimer's and Parkinson's disease, and decreased RNase oxidation activity (Kobayashi et al. 2020). Furthermore, Xue et al*.* exposed male and female C57BL6 mice to 2, 10, and 100 μg/kg/day of BPA from day 6 of gestation until weaning and at 3, 6 and 9 months of age, the neurotoxic effects were investigated. The authors reported that phosphorylated tau protein (p-tau), which is important in the stabilization of the internal microtubules in neurons, the characteristic protein of tauopathy, increased dramatically in the hippocampus and cortex at 3–9 months of age. In addition, it was also observed that GSK3β and CDK5, two critical kinases, were activated in most of the perinatal BPA exposure group, and protein phosphatase 2A (PP2A), one of the most important phosphatases, regulated the expression of p-Tau through its demethylation, methylation and phosphorylation (Xue et al. 2020). Zhang et al*.* evaluated the neurotoxic effects of the male mouse hippocampus under exposure to 0.01, 0.1 and 1 mg/L of BPA. Overall, exposure to BPA inhibited mRNA and protein expressions of oxidative phosphorylation (OXPHOs), decreased learning ability by suppressing mitochondrial OXPHOs and altered the expressions of 16 overlapping differentially expressed genes involved in Alzheimer’s disease, Parkinson’s disease, Huntington’s disease and OXPHOs in the hippocampus (Zhang et al. 2022b). In addition, the effect of vitamin E and selenium to combat the toxicity of 25 mg/kg body weight of BPA on the spinal cord and submandibular glands was evaluated in adult male 5-month-old albino rats (*Rattus norvegicus*). BPA caused a neurotoxic effect in the spinal cord of rats, nerve fibre degeneration with axonal disappearance of the white matter, GFAP proliferation and high level of MDA in the spinal cord and degeneration of the acinar cells of the submandibular gland and duct system. (Bashir et al. 2022).

#### Effects in ewe models

##### Specific brain area modifications

Regarding the effect of BPA on specific brain area modifications, along with neurobehavioural and cognitive disorders, one study has been performed on pregnant ewes. The authors exposed these animals to 5 µg/kg of BPA to evaluate the impact on foetal brain development in a region-specific way. The metabolic pathways excitatory and inhibitory amino-acid, cholinergic, energy, and lipid homeostasis pathways were particularly affected, which might be a factor in the neurobehavioural and cognitive disorders. The dorsal hippocampus, the cerebellar vermis, the dorsal hypothalamus, the caudate nucleus, and the lateral section of the frontal cortex were affected by BPA (Guignard et al. 2022).

In conclusion, in vivo research supports in vitro studies. These studies showed that BPA can cause changes in the neurological system, like structural and molecular alterations in cells and specific brain sections, mostly in the hippocampus and the cortex (Table 2). In the articles reviewed detrimental effects on neurotransmitters and modifications of expression and activity in important genes and proteins, excitotoxicity, oxidative stress, neuroinflammation, and damaged blood–brain barrier function have been detected. These modifications can lead to neurodevelopment and neurological implications and pathologies such as deteriorated cognitive functions and cholinergic system, impaired spatial learning, and memory capacity, neuroinflammation, stroke-related problems, neurodegenerative diseases (e.g., Alzheimer’s, Parkinson’s, and Huntington´s), and neuropsychological or neurobehavioural (social and emotional) disorders.

**Table 2:** Summaries of the disruptive effects of BPA in the animal in vivo studies

Topic		Animals/Organs/Cells	Drugs	Concentration	Results	References
Worm models	Neurodevelopment	Asexual Smed CIW4 worms	BPA BPF Bisguaiacol Estradiol	1.998 mg/L	BPA had profound, nonestrogenic effects on planarian neuroregeneration BPA shows a severe lack of connection between nerve cords and longitudinal spreading of the brain primordia into the head periphery	Morris et al. (2022)
Fish models	Morphological changes	Zebrafish embryos (*Danio rerio*)	BPA BPS 2,4-BPS TGSA D-8	100 nM and 100 μM	Induced phenotypical malformations at 100 μM No effects on heartbeat, development, or behaviour at 100 nM of BPA	Björnsdotter et al. (2017)
		Zebrafish male and female	BPA Quercetin	BPA (17.52 μM) Quercetin (2.96 μM)	BPA-induced augmented oxidative stress and pyknosis in the periventricular grey zone region of zebrafish brain BPA altered the usual scototaxis behaviour of zebrafish BPA increased lipid peroxidation and protein carbonylation BPA altered the brain antioxidant status BPA decreased the level of glutathione, glutathione reductase, glutathione-s-transferase, and superoxide dismutase	Sahoo et al. (2020)
		Adult zebrafish (*Danio rerio*)	BPA Taurine	4 mg/L (17.52 μM)	Augmented oxidative stress Neuronal pyknosis and chromatin condensation	Pradhan et al. (2021)
	Neurodevelopment	Adult wild-type zebrafish	NPPs BPA	0.78 and 1 μg/L BPA or NPPs (1 mg/L) or BPA (1 μg/L) and NPPs (1 mg/L)	BPA shown accumulation in the viscera, gill, head, and muscle Increased 2.2-fold and 2.6-fold in BPA uptake in the head and viscera in co-exposure of NNP and BPA Down-regulation of Syn2α protein in BPA treatment Induction of myelin basic protein expression Co-exposure of BPA and NPPs contribute to dopaminergic neurotoxic effect BPA and NPPs caused AChE activity inhibition	Chen et al. (2017)
		AB strain adult zebrafish	BPA n-TiO2	BPA (2 and 20 μg/L) and n-TiO2 (100 μg/L)	Decreased thyroid hormones by co-exposure Exposure to 20 μg/L BPA decreased the thyroxine concentration in adult plasma	Guo et al. (2019)
		Zebrafish (wild type AB strain and transgenic lines Tg(cyp19a1b:GFP)	BPA, BPS, BPF, BPAF, BPAP and 17α- ethinylestradiol	1 and 0.1 µM	Acceleration of hatching rate of embryos High levels of AroB protein expression in multiple brain areas	Coumailleau et al. (2020)
		Zebrafish embryos	n-TiO2 BPA	n-TiO2 (100 μg/L) BPA (1, 4 and 20 μg/L)	Decreased transcription level of α1-tubulin, myelin basic protein and Syn2a in an exposure concentration-dependent manner, with significant differences at 4 and 20 μg/L BPA groups Reduced protein expressions of α1-tubulin, myelin basic protein and Syn2a were observed in 4 and 20 μg/L groups	Fu et al. (2020)
		zebrafish embryos Tg(HuC:GFP) zebrafish	BPA BPS	0.01, 0.03, 0.01, 0.3, 1 μM BPA/BPS	qRT-PCR results showed that BPA and BPS could interfere with the normal expression of development-related genes vegfa, wnt8a, and mstn1 at the developmental stages Expression of neurodevelopment-related genes (ngn1, elavl3, GFAP, α1-tubulin, myelin basic protein, and gap43) were upregulated in BPA and BPS treatments, except for the downregulation of myelin basic protein and gfap elicited by BPA at 0.03 μM and 0.3 μM, respectively	Gyimah et al. (2021)
		Zebrafish embryos	Estrone BPA	Estrone (0.01, 0.1, 1, 10, or 100 nM) or BPA (1, 10, 100, 1000, or 10,000 nM)	Estrone and BPA exert phenotypic or transcriptomic responses as low as 1 nM Estrone or BPA 0 to 5 days after fertilization exposure causes non-monotonic transcriptomic changes BPA 4 to 5 days after fertilization exposure causes hypoactivity and expression of neurological genes BPA at concentrations equal or above 100 nM affected locomotive behaviour and changed the expression of both estrogenic and non-estrogenic genes	Wu et al. (2021)
	Neurobehaviour	Adult male zebrafish (*Danio rerio*)	BPA	50—500 ng/L (7 weeks)	Induced physiological abnormality Disturbed courtship behaviour	Li et al. (2017a)
		Adult male zebrafish	BPA	50—500 ng/L (7 weeks)	Disturbed colour discrimination Alleviated anxiety behaviour Altered the ability to adapt to the environment	Li et al. (2017b)
		Embryos and larval zebrafish	BPA TBBPA	1 nM to 10 μM BPA 150 pM to 1.5 μM TBBPA	10 μM BPA induced hyperactivity, hypoactivity, or had no behavioural effect	Fraser et al. (2017)
		Zebrafish embryos	BPA BPS PFOS BDE-47	1 and 10 μM	Decreased frequency of spontaneous movement for the embryos exposed to BPA and BPS at both 1 and 10 μM Decreased frequency of spontaneous movement by approximately 50% for BPA Increased movement intensities by 41% at 1 μM and 19% at 10 μM for BPA Increased intervals by 110% at 1 μM and 115% at 10 μM for BPA	Zhang et al. (2021a)
		Adult zebrafish	BPA	0.25, 0.5, 1, 2, 4,8, 16, and 32 mg/L	Increased pyknotic and Hoechst positive neurons intelencephalon and diencephalon Higher concentration of BPA adversely affects the neurobehavioral response, antioxidant status, and neuromorphology Induced neuropathological manifestation leading to the development of motor dysfunction and Parkinsonism‐like neurodegenerative phenotypes	Sahoo et al. (2021)
		AB strain zebrafish (*Danio rerio*)	BPA LCC-WS-A LCC-WS-B	BPA (500 μg/L) or BPA + LCC-WS-A (1 mg/L) or BPA + LCC-WS-B (1 mg/L)	Negative effects of BPA exposure on the exercise behaviour, the catalase and superoxide dismutase activity, larval development, gene expression in zebrafish larvae, behavioural inhibition, and also prevented the expression of CNS proteins in transgenic zebrafish models	Gu et al. (2021)
		Zebrafish embryos and larvae	BPA BPF BPAF BPS	200 μg/L	Effects on embryo development at a safe dose Decreased neurotransmitters GABA Increased concentrations of glutamate and glutamine Modified levels of five dopamine neurotransmitters	Gu et al. (2022)
		Zebrafish larvae	BPA	17.52 µM	Altered scototaxis behaviour in light–dark preference test In the Novel Tank Diving Test, zebrafish spent more time in the upper zone compared to control groups, suggesting that duration spent in the upper zone of the tank increased	Thayumanavan et al. (2022)
		Zebrafish embryos/larval	BPA and C3G	600 μg/L BPA or 600 μg/L BPA + 5 μg/mL C3G or 600 μg/L BPA + 25 μg/mL C3G or 600 μg/L BPA + 50 μg/mL C3G or 50 μg/mL C3G	BPA caused effects on locomotor behaviour and changes in brain morphology in zebrafish larvae Induced the decline of GSH, SOD, GPx CAT, oxidative stress and cell apoptosis	Yang et al. (2023)
	Neurovascular and neuroinflammatory issues	Goldfish	BPA	50 μg/L	Disrupted dopaminergic processes and damage blood vessels Induced brain atherosclerosis	Wang et al. (2019c)
		Zebrafish embryos	BPA Biochanin A	1 µm of BPA	1 µm of BPA exposure elevated levels of NO and LDH, and inflammation-related expression levels while considerably decreasing SOD, CAT, GST, GSH, AChE and glutathione-related enzyme activity in the head region BPA promoted oxidative stress damage and cell death BPA reduced speed of the swimming of the larvae and impaired the locomotor activity	Haridevamuthu et al. (2022)
Flie models	Neurobehaviour	*Drosophila melanogaster* (w1118 strain and the Fragile X Syndrome (FXS) model)	BPA	0.1 and 1 mM	Promotes recurrent grooming behaviour in adults, lowers courting behaviour in larvae, inhibits axon guidance in the mushroom body, and interferes with the formation of neural stem cells in w1118 genetic strain	Nguyen et al. (2021)
	Neurodevelopment	*Drosophila melanogaster*	BPA	0.25 mg/mL BPA	BPA reduced the expression of genes involved in neurodevelopment, including as those involved in learning, memory, and synaptic development as well as orthologs of human genes connected to neurological and neuropsychiatric disorders BPA impairs associative learning BPA increased the number of axonal branches	Welch et al. (2022)
Frogs, mussels, tunicates and clam models	Morphological changes	*Rhinella arenarum* embryos and larvae	BPA	1.25 to 40 mg/L	Several morphological changes during early stages Impaired gill circulation stage	Wolkowicz et al. (2014)
		*Rhinella arenarum* embryos and larvae	BADGE	0.0001 to 15 mg/L	BADGE was more toxic to embryos than to larvae at all exposure times Cell dissociation and delayed development in embryos, and in larvae was related to neurotoxicity, as scare response to stimuli and narcotic effect	Hutler Wolkowicz et al. (2016)
		Green mussels (*Perna viridis*)	Carbamazepine (CBZ) BPA Atrazine (ATZ)	Below than 76, 1178 or 9749 ng/L	No alteration of activity of AChE Induced DNA damage	Juhel et al. (2017)
	Neurodevelopment	*Phallusia mammillata* embryos	BPA, BPE, BPF, 2,2 DPP, E2B, DES, 4OHT and PTU	0.1, 1, 5, 10, 15, 20, 25 and 30 μM	BPA was median effective in 11,8 μM and lethal in 21 μM of concentrations Impaired differentiation of the ascidian pigmented cells in micromolar doses of BPA BPA affected ascidian otolith differentiation	Gomes et al. (2019)
		Invertebrate bivalve species blood clam (*Tegillarca granosa*)	BPA Microplastics	BPA (10, 100 ng/L) or BPA (10 or 100 ng/L) and Microplastics (1 mg/L)	Expression of four immune-related genes from the NFκB signalling pathway was also found to be suppressed by the BPA and MP treatment Exposure to BPA and MPs increased in vivo contents of three key neurotransmitters (GABA, DA, and ACh) but decreased expression of genes encoding modulatory enzymes and receptors for these neurotransmitters	Tang et al. (2020)
Rodent models	Morphological changes	Adult male Sprague–Dawley rats	BPA Melatonin	BPA (50 mg/kg bw) and Melatonin (10 mg/kg bw)	Increased oxidative stress in cerebral cells Decreased glutathione levels and superoxide dismutase activity Caused upregulation of CD95-Fas, caspases-3 and 8, and p53, resulting in cerebral cell apoptosis	El-Missiry et al. (2014)
		KM male mice, aged 4 weeks	BPA	0.1, 10 and 1000 μg/mL bw/day	Increased percentages of tail DNA, tail length and tail moment in brain cells with increasing BPA exposure concentrations Low dose BPA exposure led to DNA damage in brain cell Impaired learning and memory ability in long-term BPA exposure	Zhou et al. (2017)
		Wistar rat’s offspring at postnatal day 17 from pregnant females	BPA	100 μg/L	Increased percentage of p-H2AX-positive cells within the IBA1-positive microglial cells in hippocampal dentate gyrus Induced DNA damage in immune cells at both peripheral and central level Increased number of GFAP + cells Marked decreasing trend of ERα expression Induced microglial DNA damage and astrogliosis	Di Pietro et al. (2020)
		Wistar rats (PND 21–90)	BPA	BPA (40 μg/kg body weight/day)	Reduced levels of mitochondrial biogenesis proteins (PGC1α, and TFAM) and mitochondrial import protein (GFER) Induced mitochondrial damage in neurons Reduction in GFER localization Promoted translocation of cytochrome c into the cytosol and increased cleaved caspase-3 levels triggered apoptosis	Goyal et al. (2021)
		Adult Sprague Dawley rats	BPA Melatonin	BPA (100 μg/kg BW, 1 and 10 mg/kg BW) and melatonin (4 mg/kg BW)	BPA increased oxidative stress and expression of PUMA, p53 and Drp-1, which caused apoptosis in brain tissue	Ishtiaq et al. (2021)
		Male Thy1-Cre mice and Female breeding C57BL/6 mice	BPA	0.5 mg/kg/day	Reduced the number of RV + neurons in CA3 and entorhinal cortex but not medial septum BPA decreased the percentage of CaMKIIRV + cells in CA3 and entorhinal cortex Weakened synaptic connection of upstream glutamatergic neurons and CA1 pyramidal cells Detrimental effects of BPA exposure on the excitatory neural circuit of CA3-CA1 and EC-CA1 in memory formation	Bi et al. (2021)
		Male and female C57BL/6 mice	BPA	1 mg/kg/day for 10 weeks	HDAC2 was aberrantly induced	Bi et al. (2022)
		Specific-pathogen-free adult white male Wistar rats	BPA and EGCG	250 mg/kg/day BPA or 10 mg/kg/day EGCG and 250 mg BPA/kg/day	BPA induced apoptotic and necrotic effects to hippocampal CA3 neurons Decreased hippocampal concentrations of DA, NE, 5-HT, and Ach Decreased hippocampal activity of AChE in rats pre-treated with EGCG for 2 h before BPA Showed a abnormal pyramidal cells in hippocampus Increased hippocampal Casp-3 activity	El Tabaa et al. (2022)
		Male Wistar albino rats	BPA and/or 18β-GA	BPA (250 mg/kg) or BPA (250 mg/kg) and 18β-GA (50 or 100 mg/kg)	BPA increased MDA levels BPA increased levels of NF-κB and TNF-α BPA activated JAK1/STAT1 signalling pathway and levels of p38 MAPK and JNK BPA led to ER stress in brain tissue and up-regulated the levels of ATF-6, PERK, IRE1 and GRP78 BPA increased mRNA levels of Bax and caspase-3, reducing Bcl-2 levels	Caglayan et al. (2022)
	Neurodevelopment	NMRI mice	BPA	0.32, 3.2 or 4.8 mg BPA/kg body weight	Postnatal exposure caused adult disturbances in spontaneous behaviour and cognitive function Behavioural disturbances dose-dependent, long-lasting, and irreversible Postnatal exposure affected cholinergic system	Viberg et al. (2011)
		NMRI mice	BPA	0.32, 3.2 or 4.8 mg BPA/kg body weight	Postnatal exposure during the peak of rapid brain growth induced adult neurotoxic effects Increased SYP levels in cerebral cortex Decreased levels of CaMKII in cerebral cortex and hippocampus	Viberg and Lee 2012)
		Adult Wistar rats	BPA	50 µg/kg of body weight	Increased expression of P450arom and Tph2 in males Decreased 5a-R1 expression in females Identified 17 genes as potential targets of BPA of 84 genes involved in BPA	Castro et al. (2013)
		Pregnant ICR mice	BPA	0.02 or 50 mg/kg	Reduced Xist, Fmr1, Gdi1, Nlgn3, and Pak3, and increased Tsix in higher concentration Reduced Xist, Gdi1, Nlgn3, and Pak3 in lower concentration Changed ERα, ERβ, and AR expression and reduced anogenital distance and estradiol levels in both exposure groups	Kumamoto and Oshio 2013)
		BALB/cA mice, non-diabetic NOD/ShiLtJ(NOD) mice	BPA	1.4 or 13.7 mg/kg bodyweight/day (before and during the gestation) and 3.9 or 13.7 mg/kg bodyweight/day (during lactation)	Increased amount of Pax6 in the cerebellum Increased thickness of the external granule layer in 11-day-old mouse pups	Mathisen et al. (2013)
		ICR mice	BPA Diethylstilboestrol	2, 20 or 200 μg/kg/day BPA 0.5 μg/kg Diethylstilboestrol	Increased tremors in newborn infants prenatally exposed	Nagao et al. (2014)
		Wistar rats	BPA	10 μg/kg/day	Perinatal BPA exposure affected gene transcription in juvenile and adult male rats Reduced transcripts of precortical dopamine receptors in juvenile rats Produced permanent alterations in GDNF transcript levels Reduced Tph2 and increased Th transcription in adult rats	Castro et al. (2015)
		Pregnant Wistar rats	BPA	25 μg, 250 μg, 5 mg or 50 mg/kg bw/day	Female offspring experienced impaired spatial learning with exposure of 25 μg/kg bw/day of BPA	Hass et al. (2016)
		C57BL/6 J mice	BPA	40, or 400 μg/kg bw/day	Reduced spine densities in the hippocampal CA1 Decreased basal dendrite length and branching number Disrupted hippocampal CA1 neuronal morphology during development	Kimura et al. (2016)
		NCTR Sprague–Dawley dams	BPA	2.5, 25, 250, 2500, or 25,000 μg/kg bw/day	Disrupted transcriptome of the neonate amygdala by prenatal BPA exposure Make female amygdala more vulnerable in foetal development Affect signalling pathways for oxytocin, vasopressin, and estrogen	Arambula et al. (2018)
		Male Swiss albino mice weighing 20–30 g	BPA *Withania somnifera* (Ws)	BPA (50 μg/kg bw/day) or BPA with given Ws root extract treatment (100 mg/kg bw/day)	Increased level of MDA, and increased lipid peroxidation level Impaired spatial memory and short-term spatial memory Induced oxidative stress	Birla et al. (2019)
		Sprague–Dawley (SD) rats	BPA	BPA (0.5, 50, or 5000 μg/kg bw/day)	Impaired learning and memory ability Impaired hippocampal dendrites and spines Disrupted homeostasis of hippocampal neurotransmitters Neurotoxic effects of BPA exposure for rats are dependent on gender	Zhang et al. (2019)
		KM male and female mice	BPA	0.5, 50 or 5000 μg/kg bw	Maternal BPA exposure impairs the spatial learning and memory ability Maternal BPA exposure alters hippocampal neurotransmitter levels Maternal BPA exposure decreased neuron quantities and dendritic spine density across generations	Zhang et al. (2020)
		Adult male Wistar rats	BPA Curcumin	BPA (40 μg/kg body weight) or Curcumin (20 mg/kg body weight) or BPA (40 μg/kg body weight) and Curcumin (20 mg/kg body weight)	BPA induced reduction in the front of oligodendrocyte progenitor cells in the hippocampus, increased apoptosis, caused a decline in oligodendrocyte differentiation, stemmed from alteration in expression profiles of myelin-specific genes and protein levels, regulated expression down from Notch pathway genes inspired the number of axons and the ability to learn and remember Immunohistochemical analysis showed a decrease in the co-localization intensity of Olig2/myelin basic protein + and myelin basic protein/NF + in the brain hippocampal region	Tandon et al. (2020)
		Adult male albino rats	BPA Lycopene	BPA (50 mg/kg bw) or Lycopene (10 mg/kg bw) or Lycopene (10 mg/kg bw) and BPA (50 mg/kg bw)	BPA treatment provoked a significant reduction in the hippocampal GSH level by 53%, along with a fivefold rise in the hippocampal MDA level The BPA group showed decreased the hippocampal TrKB, MAPK, ERK1/2, and CREB protein expression by 75%, 83%, 82%, and 70%, respectively, along with a decline in the hippocampal BDNF level by 46% BPA administration reduced the AChE activity in the hippocampus by 50% BPA caused upregulation in the hippocampal proapoptotic gene Bax (fivefold), in addition to rush hippocampal Casp-3 activity (fourfold), accompanied by a decrease in the hippocampal antiapoptotic gene Bcl-2 (4.5-fold)	El Morsy and Ahmed 2020)
		C57BL6J mice	BPA	200 mg/kg bw/day	Differential expression of 43 small RNAs Target mRNAs closely matched those expressed by the brain and thymus	Mao et al. (2021)
		Adult female Swiss albino rats	BPA NK γ-IR	NK (720 FU/kg BW/day) or BPA (100 mg/kg BW/day) or NK (720 FU/kg BW/day) and BPA (100 mg/kg BW/day) or γ-IR (3 Gy/week) or γ-IR (3 Gy/week) and NK (720 FU/kg BW/day)	BPA increased levels of the misfolded proteins Aβ and p-tau BPA induced cognitive deficits and learning-memory impairment in offspring mice BPA decreased acetylcholine transferase activity	Elbakry et al. (2022)
		Male KM mice	BPA	0.05, 0.5, and 5 mg/kg body weight	Negative effects on spatial learning and memory as well as alterations in the expression of miRNAs in the hippocampus 17 miRNAs showed altered expression levels	Luo et al. (2023)
	Specific brain area modifications	Adult male Wistar albino rats	BPA	10 and 25 mg/kg	Increased excitatory and decreased inhibitory amino acids in cortex Increased lipid peroxidation, nitric oxide Reduced glutathione Increased excitatory and inhibitory amino acid neurotransmitters Increased hippocampal lipid peroxidation and reduced glutathione Increased cortical and hippocampal AchE activity	Khadrawy et al. (2016)
		Adult male Wistar rats	BPA	0.5, 5 and 50 mg/kg	Lipid peroxidation in 0.5 and 5 mg/kg was higher than the control group Low doses have showed some pro-oxidant effects 10 proteins expression altered by BPA in brain cortex Increased by 80% in pyruvate kinase M2 levels Increased pyruvate kinase M2 levels expression in some cancer cells, resulting in accumulation of Phosphoenolpyruvate	Tavakkoli et al. (2020)
		Wistar rat’s offspring at postnatal day 17 from pregnant females	BPA	100 μg/L	Enlarged lateral cerebral ventricles in lactating and weaned BPA-exposed animals Affected expression of inflammatory cytokines, Sirt1, its natural antisense long non-coding RNA (Sirt1-AS LncRNA) and histone deacetylase 1 (HDAC1)	Santoro et al. (2021)
		Juvenile PND20 (pre-weaning; age of 20 days) male Sprague Dawley rats	BPA Astragaloside IV *A. spinosus saponins*	BPA (125 mg/kg/day) or Astragaloside IV + BPA (80 mg/kg/day) or A. spinosus saponin + BPA (100 mg/kg/day)	Increased level of NO and decreased level of glutamate, glutamine, glutaminase and glutamine synthetase BPA decreased BDNF concentration in the hippocampal and striatal regions and affect it in the prefrontal cortex BPA upregulated NR2A mRNA expression levels in prefrontal cortex and hippocampus and downregulated the striatum NR2B mRNA expression level downregulated in prefrontal cortex and hippocampus regions and upregulated in striatum brain region BPA induced a marked DNA damage	Essawy et al. (2021)
		Sprague–Dawley rats	BPA DEHP	BPA (50 mg/kg/day) or BPA (50 mg/kg/day) and DEHP (30 mg/kg/day)	BPA and/or DEHP led to apoptosis and histopathologic alterations in the hippocampus Changed the total GSH and MDA levels and also in AchE activity	Yirun et al. (2021)
		CD-1 mice	BPA	2.25 μg BPA/kg bw/day	Structural alterations in brain regions including the superior olivary complex and bed nucleus of stria terminalis with larger effect sizes	Zheng et al. (2022)
		Two-month-old male Wistar rats	BPA and Melatonin	1 mg/kg bw BPA with or without 20 or 40 mg/kg bw MEL	BPA exerted an inhibitory effect on general locomotion and induced in the frontal lobe and the hippocampus the activation of MAPK After BPA treatment, MCP1 expression was reduced and the expression of pNFkB was enhanced BPA promoted cellular oedema, necrosis, and a polymorphic cellular inflammatory infiltration that increased the intercellular spaces	Sevastre-Berghian et al. (2022)
	Neurobehaviour	Male Wistar rats	BPA 3-hydroxybisphenol A Bisphenol A 3,4-quinone	20 ng, 0.2, 2 and 20 μg	Hyperactivity at 4–5 weeks of age in lowest BPA concentration BPA increased activity in the nocturnal phase by 1.3 times	Ishido et al. (2011)
		CD-1 male mice	BPA	50 mg BPA/kg diet per day	Increased anxiety-like behaviour Reduced AChE activity in the hippocampus	Luo et al. (2013)
		BALB/c mice	BPA	200 μg/kg per day	Prenatal exposure induced long-lasting DNA methylation changes in the transcriptionally relevant region of the BDNF gene in the hippocampus and blood	Kundakovic et al. (2015)
		NCTR Sprague–Dawley rats	BPA Ethinyl Estradiol	2.5, 25 or 2500 µg/kg body weight BPA 0.5 μg/kg bw/day ethinyl estradiol	Differences between BPA-exposed and ethinyl estradiol exposed groups were identified for several endpoints No consistent effects of BPA observed for any endpoint	Rebuli et al. (2015)
		Eight-week-old male C57BL/6 J mice weighing between 25 and 30 g	BPA α-Lipoic acid	10 mg/kg BPA 50 mg/kg α-Lipoic acid	Increased activity of monoamine oxidase in the mouse brain in BPA group BPA decreased AChE activity, CAT activity, GST activity and GSH content in brain BPA increased lipid peroxidation and the content of protein oxidation Combined treatment showed a significantly higher level of GSH	Khan et al. (2018)
		C57BL/6 mice	BPA	5 mg BPA/kg	259 genes overrepresented in categories related to mating, cell–cell signalling, behaviour, neurodevelopment, neurogenesis, synapse formation, cognition, learning behaviours, hormone activity, and signalling receptor activity, among other things, in the mice exposed to BPA	Henriksen et al. (2020)
		C57/BL6 mice	Mixture with BPA, DEP, DBP, BBzP, DIDP/DPHP, 3-PBA, p,p’-DDE and TCP	0.0075, 1.500, 14.999 and 74.994 μg / Kg BW BPA	Mixture increased active coping during swimming stress Mixture increased locomotion and decreased social interaction in male offspring Mixture altered the expression of all examined genes (Nr3c1, Crh, Fkbp5, Oxt, and Esr2) in the hypothalamus, primarily in male progeny	Repouskou et al. (2020)
		Wistar albino rats	BPA	5 mg/kg body weight	Caused anxiety-like behaviour Decreased serum GABA levels Reduced dendritic spine counts and astrocytes numbers	Singha et al. (2021)
		Pregnant Sprague Dawley rats	NeuroMix (A1221, BPA, BPS, DEHP, DBP, PFOS, PBDE-47, PCB-153 and Vinclozolin)	2.5 µg/kg BPA	Adult females were the only ones affected by NeuroMix's effects on anxiety-like, social, and mate preference behaviours Stress had more of an impact on male than female Many relationships between NeuroMix and stress were discovered, particularly for brain gene expression and partner choice behaviour	Gore et al. (2022)
		Mice (C57BL/6N)	BPA or DMSO (control)	50 µg/kg BPA or 0.1% DMSO for 2 months	BPA resulted in elevated anxiety behaviour BPA disrupted memory processing	Hyun et al. (2022)
	Stroke-related problems	Adult male C57/BL6 mice	BADGE Bexarotene BR1211	30 mg/kg BADGE 0.5, 5 or 25 mg/kg Bexarotene 1 mg/kg BR1211	Pharmacological blockade of PPARγ (with BADGE) reverted reduction of brain infarct volume and oedema induced by bexarotene	Certo et al. (2015)
		Male Sprague Dawley rats	Ursolic acid BADGE	MCAO/R + 5, 10 or 20 mg/kg Ursolic acid) or (MCAO/R + 5, 10 or 20 mg/kg Ursolic acid + 30 mg/kg BADGE) or (MCAO/R + 30 mg/kg BADGE)	Elimination of the ursolic acid-induced enhancement in PPAR-γ expression BADGE blocked the levels of MMP2 and MMP9 and increased TIMP1 level, previously induced by ursolic acid	Wang et al. (2016)
		Male Sprague–Dawley rats	MIF BADGE	3 mg/kg MIF and 30 mg/kg BADGE	Increased neurological deficit scores and TNF-α, IL-1β and IL-6 levels MIF effect combined with BADGE was beneficial on neurological function and brain infarct volume	Wu et al. (2018)
	Neuroinflammation	Adult male Wistar rats	BPA L-NAME NAR	BPA (100 mg/kg) 40 mg/kg L-NAME and 100 mg/kg BPA 100 mg/kg BPA and 80 mg/kg NAR 80 mg/kg NAR and 40 mg/kg L-NAME and 100 mg/kg BPA	BPA increased motor coordination-linked behaviour BPA up-regulated proinflammatory mediators BPA increased antigen presenting cell and T-killer CD43 cell	Akintunde et al. (2020)
		Eight-week-old adult male and female C57BL/6 J mice	BPA	0.05, 0.5, 5, and 50 mg/kg BPA for 22 weeks	Induced cognitive impairment in male but not in female Increased neuroinflammation and damaged blood–brain barrier function, caused gut barrier dysfunction and altered the microbiota composition Decreased 5-HT and its metabolite levels in the serum and hippocampus	Ni et al. (2021)
		Adult male Wister albino rats	BPA or BPA and GSPE	50 mg/kg BW/day BPA or 200 mg/kg BW/day GSPE + 50 mg/kg BW/day BPA	Increased MDA and NO levels in the brain Increased serum levels of total cholesterol, triglycerides, and LDL Increased specific activity of AchE Increased levels of dopamine and serotonin Induced expression of TNF-α, COX-2 and p53 genes	Abdou et al. (2022)
		Male C57Bl/6 J mice	BPA	50 μg/kg	The anxiety-like behaviour caused by the HFD and BPA group got worse Neuroinflammation brought on by HFD was made worse by BPA, which also increased IL-1, TNF-, and monocyte chemoattractant protein MCP1 in PFC Generated monocyte recruitment and inflammation in the hypothalamus and amygdala BPA increased immune activation caused by excess lipids and caused mastocytosis in the hypothalamus	Lama et al. (2023)
	Neurodegeneration	Adult Wistar rats	BPA	40 or 400 μg/kg body weight	Early postnatal period exposure enhanced the expression and the levels of autophagy genes/proteins Downregulated mammalian target of rapamycin pathway and resulted in mitochondrial loss, bioenergetic deficits, and increased PARKIN mitochondrial translocation	Agarwal et al. (2015)
		Wistar rats	BPA	40 μg/kg body weight	Increased apoptotic cell death through inhibition of autophagy Increased levels of Drp-1 in the hippocampal tissue	Agarwal et al. (2016)
		Adult female Wistar rats	BPA Curcumin	BPA (40 μg/kg body weight) or Curcumin (20 mg/kg body weight) or BPA (40 μg/kg body weight) and Curcumin (20 mg/kg body weigh)	Downregulated expression of antiapoptotic gene Bcl-2, while upregulated Bax Increased neural degeneration	Tiwari et al. (2016)
		C57BL6 male mice	BPA	100 μg/kg/day BPA	Decreased of insulin sensitivity, and glucose transporter 1, 3 protein levels Hyperactivation of IR/AKT/GSK3β axis Increased phosphorylated tau and β-APP	Li et al. (2016)
		C57BL/6 J male mice	BPA	40 and 400 μg BPA/kg body weight	Caused oxidative stress in brain, memory loss, muscle coordination deficits, allodynia, degeneration of mature and immature oligodendrocytes, nestin, myelin basic protein, and myelin-associated glycoprotein-1 Subchronic exposure to BPA caused neuroinflammation 40 μg/kg body weight of BPA caused axonal degeneration and demyelination in oligodendrocytes and neurons	Khan et al. (2019)
		Adult female Wistar albino rats	BPA MSM γ-IR	BPA (150 mg/kg/day) or MSM (400 mg/kg/day) and BPA (150 mg/kg/day) or BPA (150 mg/kg/day) and γ-IR (3 Gy/week) or BPA (150 mg/kg/day) and MSM (400 mg/kg/day) and γ-IR (3 Gy/week)	BPA and γ-IR co-exposure induced typical hallmarks of neurodegenerative disorders like elevated oxidative stress, extensive neuroinflammation, elevated Alzheimer disease markers in cortex and hippocampus Discovered neurodegenerative lesions on histopathological examination	Abdel-Rafei and Thabet (2020)
		Male Sprague–Dawley rats	BPA Rotenone	BPA (100 mg/kg/day) or Rotenone (3 mg/kg/day)	S-nitrosylation of PDI detected in brain microsomes BPA and rotenone decreased RNase oxidation activity	Kobayashi et al. (2020)
		Male and female C57BL6 mice	BPA	2, 10, 100 μg/kg/day	Increased p-Tau in the hippocampus and cortex at 3–9 months of age Activated GSK3β and CDK5 Protein phosphatase 2A regulated p-Tau expression through its demethylation, methylation and phosphorylation	Xue et al. (2020)
		Kunming strain mice	BPA	0.01, 0.1 and 1 mg/L	Inhibited the mRNA and protein expressions of the OXPHOs Decreased learning ability through suppression of mitochondrial OXPHOs Altered the expressions of 16 genes overlapping with differentially expressed genes involved in the Alzheimer disease, Parkinson disease, Huntington disease and OXPHOs in the hippocampus	Zhang et al. (2022a, b)
		Adult male 5 months old albino rats (*Rattus norvegicus*)	BPA Vitamin E selenium	25 mg/kg body weight of BPA three times a week	BPA had a neurotoxic effect on spinal cord BPA provoked degeneration of nerve fibbers with axonal disappearance of white matter BPA promoted GFAP proliferation and high MDA level in spinal cord BPA provoked degeneration of submandibular gland’s acinar cells and duct system	Bashir et al. (2022)
Ewe model	Specific brain area modifications	Adult pregnant Lacaune ewes	BPA	5 µg/kg	Affected metabolic pathways excitatory and inhibitory amino-acid, cholinergic, energy, and lipid homeostasis pathways Affected the dorsal hippocampus, the cerebellar vermis, the dorsal hypothalamus, the caudate nucleus, and the lateral section of the frontal cortex	Guignard et al. (2022)

## Effects of BPA on Humans

Some studies positively associated an increased risk of premature complications with environmental factors, such as exposure to EDCs. For this, human studies on their effect are needed and can be divided into human in vitro studies, and epidemiological studies. In these types of studies, a more detailed link to neurological diseases, such as neurovascular, neurodevelopmental, and neurodegenerative diseases and their direct effects on humans will be seen, with some focus on Alzheimer's, Parkinson's, depression, ADHD, ASD, and some other cognitive and social disorders.

In sum, this section will discuss the main effects of BPA in humans through in vitro studies and the epidemiological evidence supporting a relationship between BPA and neurological complications or diseases.

### Human in vitro studies

The in vitro effects of BPA on human brain cell lines will be reviewed, namely human neural progenitor cells, SH-SY5Y, human glutamatergic neurons, human cortical neurons, human embryonic stem cells, Human peripheral blood mononuclear cells, IMR-32 from males and SK-N-SH from females.

#### Nervous system homeostasis effects

Huang B et al*.* hypothesized that maternal exposure to low doses of BPA during gestation may decrease the expression of insulin-like growth factor 1 (IGF-1), which is an essential animal multifunctional peptide that may play an important role in CNS homeostasis. The authors observed in human embryonic stem cells (hESCs)-derived embryoid bodies (hEBs), that BPA exposure-induced expression of IGF-1 insufficiency, and this IGF-1 expression can be related to inducing Parkinsonism (Huang et al. 2014). Additionally, the same authors later, in the same cells, demonstrated that this EDC can repress the expression level of IGF-1, marker genes for ectoderm, neuron progenitor cells and dopaminergic neurons. Plus, this study, has shown remarkably that BPA represses neuronal differentiation through downregulating IGF-1 expression (Huang et al. 2017).

#### Morphological changes

Concerning the morphologic alterations, Wu et al*.* analysed the BPA exposed in mitotic human neural progenitor (hNP) cells for 14 days. As a result, a decreased cell percentage by 45.2% at the highest dose assessed occurred. At 10 μM of BPA, significant reductions of 29.0%, 13.9% and 42.8% were observed for neurite length per neuron, neurites per neuron, and branch points per neuron respectively (Wu et al. 2016). Furthermore, Wang et al*.* investigated the chronic exposure to BPA (0.1, 1.0 and 10 μM) in human glutamatergic neurons (hGN), derived from hESC, for 14 days. BPA caused an axon growth reduction in a concentration-dependent manner, and a postsynaptic density protein-95 (PSD-95), SYP and α-amino-3-hydroxy-5-methyl-4-isoxazole-propionic acid receptors (AMPARs) expression were increased, that are linked to deteriorated dendritic spines and caused neuronal apoptosis. Moreover, the authors found that the BPA-induced adverse effects were due to increased production of reactive nitrogen species (RNS), ROS and an excessive influx of Ca^2+^ (Wang et al. 2019a). Further on, by the same group, human cortical neurons (hCNs) were incubated with BPA, 0.1, 1 and 10 μM for 14 days, concluding that there was a positive regulation of *N*-methyl-d-aspartate receptors and a disruption of intracellular calcium homeostasis. These authors also saw mitochondrial dysfunction, endoplasmic reticulum stress, neural apoptosis, and neuronal network damage (Wang et al. 2019b). On the other hand, Liang et al*.* incubated hESCs and NSCs to BPA and derivates. Therefore, BPA and derivates were shown to be cytotoxic at micromolar concentrations in both cell types. At nanomolar concentrations, BPA decreased axon length. Thus, the authors found that BPA substitutes may not be as neurologically safe as initially proposed (Liang et al. 2020). Furthermore, in two different cell lines, the research group of Wang C investigated the neurotoxic effects of BPA, BPS and BPB. They exposed the IMR-32 cell line of males and the SK-N-SH cell line of females, at concentrations of 1 nM, 10 nM, 100 nM, 1 μM, 10 μM and 100 μM for 24 h. The data demonstrated that bisphenols exposure can promote ROS production and increase MDA levels, SOD levels reduction, leading to oxidative stress and apoptosis in human neuroblastoma cell lines. Especially, BPA resulted in the loss of mitochondrial membrane potential (MMP) and the protein expression levels of Bak-1, Bax, cytochrome c and caspase-3 were up-regulated, but Bcl-2 was down-regulated significantly (Wang et al. 2021).

#### Genetic alterations

It has also been observed in several studies that exposure to BPA can alter the expression of important proteins for healthy brain function. In this regard, the group of Wang T used the SH-SY5Y cell line, which is a human neuroblastoma cell line, and incubated with BPA at 2, 20, 200, and 2000 nM/L. As a consequence, an inhibition of physiological p-IR Tyr1355 tyrosine phosphorylation, p-IRS1 tyrosine 896, p-AKT serine 473, and p-GSK3α/β serine 21/9 was observed, as well as increased phosphorylation of IRS1 Ser307, and there was a substantial increase in BACE-1, APP, β-CTF, α-CTF, Aβ 1-42 and phosphorylated tau proteins (S199, S396, T205, S214 and S404), which are pathological proteins associated with Alzheimer's disease (Wang et al. 2017). Pistollato et al*.* exposed human induced pluripotent stem cell (hiPSC)-derived NSCs to different chemicals, namely BPA, to evaluate the effects of acute (3 days) and repeated doses (14 days) As a result, there was a slight decrease in total PSD-95 levels and a trend toward an increase in SYP in neurites was seen after 3 days with 28.96 μM of BPA, while 14-day exposure caused a slight decrease in PSD-95 levels in neurites with 12.74 μM of BPA. There was an increase in the number of neurites after 3 days with 12.74 μM of BPA, while the number of branch points began to decrease after 14 days. Finally, the ratio between BDNF levels in neurites and cell body decreased at 12.74 μM BPA (Pistollato et al. 2020). Subsequently, the same group performed a similar experiment adding some other chemicals. Therefore, after 14 days of treatment, most chemicals induced significant positive regulation of BDNF levels, especially at the highest concentration assessed, with BPA being the main driver of positive BDNF regulation in the mixture between chlorpyrifos, lead, 2,2′4,4′-tetrabromodiphenyl ether and ethanol. Also, it was observed that the number of neurites per neuron, ethanol resulted as the chemical that contributed most to this mixture effect, while BPA and lead induced opposite effects (Pistollato et al. 2021). In the same sense, Engdahl et al*.* investigated whether BPA affects the function of the breast cancer resistance protein (BCRP), which is one of the major ATP-binding cassette (ABC) transporter proteins that take part in multi-drug resistance in cancer therapy. To shield the brain from a variety of chemicals, the BCRP is an important efflux transporter in the blood–brain barrier, and the authors in this study concluded that BPA significantly inhibited the transport function of BCRP (Engdahl et al. 2021). Furthermore, Chesnut et al*.* exposed neural progenitor cells (NPCs) derived from induced hiPSCs to BPA (50 and 100 μM) and observed a reduction in myelination (Chesnut et al. 2021).

#### Oxidative stress and stimulation of cytokine production

Regarding the relationship between the effects of BPA on Alzheimer’s, Parkinson’s, Dementia and neuroinflammation, the oxidative stress and stimulation of cytokine production were analysed. From in vitro incubation, human serum was incubated with 1 and 10 mg/mL of BPA. The serum was obtained from 94 random human donors aged 18 to 65 years. Kharrazian D et al*.* aimed to investigate associations between unconjugated BPA bound to human serum albumin antibodies (BPA-HSA) and alpha-synuclein antibodies and between IDP antibodies and alpha-synuclein antibodies, demonstrating that there were significant associations and risks between antibodies formed against BPA-bound-to-albumin antibodies and antibodies formed against alpha-synuclein, which with an abnormal deposition, plays a central role in the pathogenesis of disorders such as Parkinson's disease and Dementia (Kharrazian et al. 2019). Moreover, the group of Arita Y et al*.* exposed placental explant cultures to varying doses of BPA in the presence or absence of *Escherichia coli* colonies. As a result, it increased IL-1 and IL-6 production under basal conditions in a dose-dependent manner, 8-IsoP expression at 10 and 100 nM and BDNF synthesis at 1000 and 10,000 nM, but inhibited sgp130, a soluble receptor that reduces IL-6 bioactivity (Arita et al. 2019). Furthermore, in human peripheral blood mononuclear cells (PBMC), Di Pietro et al*.* saw that BPA-induced cell proliferation in the S phase of the cell cycle and caused chromosomal fragmentation affecting the DNA damage checkpoint. BPA further induced ɣH2AX activation in TCD4þ and TCD8þ lymphocytes in human proliferation and modulated the expression of ERα and ERβ. Finally, BPA exposure also caused microglial DNA damage and astrogliosis, thus it can be concluded that BPA can induce neurotoxicity in this cell type (Di Pietro et al. 2020). Wang C et al*.,* using IMR-32 and SK-N-SH human neuroblastoma cells, aimed to investigate the BPA effect in EGCG, Z-YVAD-FMK (a caspase-1 inhibitor) and ICI182,780 (an ER inhibitor) modulators. Experimentally, BPA increased the mRNA levels of IL-18, ASC, gasdermin D (GSDMD) and the protein levels of NLRP3 (linked to inflammatory diseases), and caspase-1 in both cell lines in a non-linear manner. BPA exposure further significantly increased reactive oxygen species production, and promoted apoptosis and LDH release, with reduced cell viability and mitochondrial membrane potential, in both cell lines. Thus, the authors concluded that BPA could induce pyroptosis in neuroblastoma cells through the NLRP3/caspase-1/GSDMD pathway, mediated by ER (Wang et al. 2022a).

In summary, human in vitro studies have shown both cellular and structural human effects on the neurological system. The BPA exposome has been associated with neurodegenerative diseases such as Parkinson's, Dementia and Alzheimer's (Table 3). The human studies analysed in this review focused on studying the effects of BPA exposure on diverse types of neurons, and neuronal damage, apoptosis effects and also disruption of intracellular Ca^2+^ homeostasis were observed. Wang C et al*.* showed that exposure to BPA in IMR-32 and SK-N-SH human neuroblastoma cells increased mRNA levels of IL-18, ASC, GSDMD, protein levels of NLRP3 and caspase-1, associating them with inflammatory diseases and pyroptosis in neuroblastoma cells. Also in this study, exposure to BPA caused a significant increase in ROS and promoted apoptosis and LDH release. In addition, effects such as decreased axon length, microglial DNA damage, astrogliosis, BCRP inhibition, and significantly reduced myelination were mentioned by the other studies.

**Table 3:** Summaries of the disruptive effects of BPA in in vitro studies using human cell lines

Topic	Animals/Organs/Cells	Drugs	Concentration	Results	References
Nervous system homeostasis	Embryoid body derived from hESCs	BPA	–	Maternal exposure to low-dose BPA decreased IGF-1 expression	Huang et al. (2014)
	hEBs	BPA	1 μM	BPA exposure repressed the expression level of IGF-1 and marker genes for ectoderm, neuron progenitor cells, and dopaminergic neurons BPA represses neuronal differentiation through downregulating IGF-1 expression	Huang et al. (2017)
Morphological changes	hNP cells	Bis-1 BPA Acetaminophen Testosterone β-estradiol	0.01, 0.1, 1, 10 mM BPA	BPA exposure decreased total cell count by 45.2% at the highest dose tested BPA exposure decreased neurite length per neuron, neurites per neuron, and branch points per neuron	Wu et al. (2016)
	hGNs derived from hESCs	BPA	0.1, 0.5, 1, 5 and 10 μM	BPA upregulated PSD-95, Syn and AMPARs expression and induced dendritic spines deterioration and neuronal apoptosis BPA-induced adverse effects are due to an increase production of RNS and ROS and excessive Ca2 + influx	Wang et al. (2019a)
	human embryonic stem cells-derived human cortical neurons (hCNs)	BPA	0.1, 1 and 10 μM BPA	BPA upregulated NMDARs and disturbed intracellular calcium homeostasis BPA induced mitochondrial dysfunction and endoplasmic reticulum stress BPA induced neural apoptosis and neuronal network damage	Wang et al. (2019b)
	hESCs and NSCs	BPA BPS BPF BPE BPZ BPB BPAF	0.001, 0.01, 0.1, 1, 5, 10, 25, 50, 100, 150, 200, 250 and 300 μM BPA	BPA is cytotoxic at micromolar concentrations in hESCs and NSCs Nanomolar concentrations of bisphenols decrease neurite length in neuron-like cells BPA replacements may not be as safe as initially proposed	Liang et al. (2020)
	2 Human neuroblastoma cell lines IMR-32 cell line from male, SK-N-SH cell line from female)	BPA BPS BPB	1 nM, 10 nM, 100 nM, 1 μM, 10 μM, and 100 μM for 24 h	BPs exposure promoted ROS production and increased level of MDA while decreased levels of SOD BPs lead to oxidative stress and apoptosis in human neuroblastoma cell lines BPA resulted in loss of MMP to accelerate BPA-induced apoptosis Male was more susceptible and vulnerable to BPs exposure than female	Wang et al. (2021)
Genetic alterations	SY5Y cells	BPA	2, 20, 200, and 2000 nM/L BPA	Inhibition of physiological p-IR Tyr1355 tyrosine, p-IRS1 tyrosine 896, p-AKT serine 473 and p-GSK3α/β serine 21/9 phosphorylation, as well as the enhancement of IRS1 Ser307 phosphorylation BACE-1, APP, β-CTF, α-CTF, Aβ 1–42 and phosphorylated tau proteins (S199, S396, T205, S214 and S404), were substantially increased after BPA exposure	Wang et al. (2017)
	human induced pluripotent stem cell (hiPSC)-derived neural stem cells (NSCs)	lead(II) chloride BPA methylmercury chloride chlorpyrifos valproic acid PCB138	0.29, 12.74 and 28.96 μM	3 days BPA exposure decreased PSD95 levels and increase SYP in the neurites 14 days BPA exposure caused a slight decrease of PSD95 at neurite levels 3 days BPA exposure increased the number of neurites 14 days BPA exposure decreased the number of branch points Ratio between the levels of BDNF in the neurites and in the cell body decreased at 12.74 μM BPA	Pistollato et al. (2020)
	Human iPSC derived NSC	lead(II) chloride BPA methylmercury chloride chlorpyrifos valproic acid PCB138 2,2′4,4′-tetrabromodiphenyl ether, Ethanol, Vinclozolin and 2,3,7,8-tetrachlorodibenzo-p-dioxin	1.59–12.74 μM BPA	After 14d treatment, majority of single chemicals induced a significant upregulation of BDNF levels (BPA, CPF, Lead, Methyl-Hg and Vincl) especially at the highest tested concentration BPA resulted as the major driver of BDNF upregulation in the 5-Sim mixture By looking at the number of neurites per neuron upon 5-Sim mixture exposure, EtOH resulted as the chemical contributing the most to this mixture effect, while BPA and Lead induced opposite effects	Pistollato et al. (2021)
	The blood–brain barrier model of iPSC-derived human-induced brain microvascular endothelial-like cells (BMECs)	BPA BPS BPF	500 nM	BPA inhibited the transport function of BCRP	Engdahl et al. (2021)
	Neural progenitor cells	Cuprizone Methylmercury chloride BPA TDCPP	BPA (50 and 100 μM)	Exposure to BPA resulted in significantly, although not concentration-dependent, reduced myelination, and led to statistically significant reduction of PLP1 in total fluorescence	Chesnut et al. (2021)
Oxidative stress and stimulation of cytokine production	Sera from 94 random human donors aged 18 to 65	BPA HSA	1 and 10 mg/mL of BPA	Significant associations and risks between antibodies to BPA–HSA adducts and PDI antibodies for developing alpha-synuclein antibodies	Kharrazian et al. (2019)
	Placental explant cultures	BPA	0–10,000 nM	Increased IL-1 and IL-6 production under basal conditions in a dose-dependent manner, but inhibited sgp130 Increased 8-IsoP expression by 10 and 100 nM and BDNF synthesis by 1000 and 10,000 nM	Arita et al. (2019)
	Human peripheral blood mononuclear cells (PBMC)	BPA	5, 10, 25, 50, 100 or 200 nM 25, 50, 100 or 200 μM	Induced the accumulation of cells in S-phase of the cell cycle in human PBMC Caused chromosome fragmentation by affecting DNA damage checkpoint in human PBMC Induced ɣH2AX activation in human proliferating TCD4þ and TCD8þ lymphocytes Modulated ERα and ERβ expression in a sex specific manner in human PBMC Induced microglial DNA damage and astrogliosis	Di Pietro et al. (2020)
	Human neuroblastoma cell lines IMR-32 and SK-N-SH	BPA EGCG Z-YVAD-FMK ICI182.780	0, 1, 10 and 100 nM 1, 10 and 100 μM	Increased mRNA levels of IL-18, ASC, GSDMD and protein levels of NLRP3, caspase-1 and GSDMD in both cell lines in a nonlinear manner Increased ROS production, LDH leakage and apoptosis, with reduced cell viability and mitochondrial membrane potential, in both cell lines	Wang et al. (2022a)

### Epidemiological studies

Human exposure to BPA has been analysed mainly in the early stages of development, such as pregnancy and the prenatal period, when individuals are most susceptible to its effects, both in vitro and in vivo, but it is epidemiologically where the negative effects of both BPA and other EDCs on the human body are most accurately perceived, in this case relating to brain disorders (Abrantes-Soares et al. 2022; Thayer et al. 2015). Thus, this topic mostly relates prenatal exposure to BPA to neurological, and neurodevelopmental disorders and neurological pathologies, like ADHD, ASD, and even Stroke.

#### Neurodevelopmental problems

Regarding neurodevelopmental problems caused by BPA exposure, we found some epidemiological studies reporting changes in brain functions and cognitive development. The group of Myridakis A evaluated the exposure of preschool children to BPA, phthalates, and parabens. This study was part of the “RHEA” project, which is a pregnancy cohort, prospectively examining a cohort of pregnant women between February 2007 and February 2008 and their children in Heraklion, Crete, Greece. Although during the study, the link of parabens to high BPA exposure was studied since they can be found in food, they do not share the same source of exposure. Yet, a negative correlation was seen between age and high BPA exposure (Myridakis et al. 2016). Braun et al*.* investigated the relationship between prenatal urinary BPA metabolite and visuospatial abilities in a prospective cohort in Cincinnati, HOME study of 198 mother–child pairs. However, maternal urinary BPA concentrations during pregnancy, but no association was observed (Braun et al. 2017a). Moreover, Martinez MA et al*.* estimated prenatal exposure to BPA and DEHP through food consumption of pregnant women, as part of the European “HEALS” project, living in the municipality of Tarragona (Spain). As a result, plasma concentrations of free BPA were observed in women characterized by transient peaks due to meals, and foetuses showed high basal sustained BPA concentrations due to low or no metabolic activity. Thus, the authors concluded that these EDCs may have a major effect on organ development in young children or the foetus, including brain development (Martínez et al. 2017). On the other hand, Lin CC et al*.* investigated the association between cord blood BPA levels and neurodevelopmental outcomes at 2 and 7 years of age. In the sample of 356 mother–child pairs from the Taiwan Birth Panel Study, BPA was detected in cord blood in 55.9% of the children. In boys, there were significant associations between the full-scale intelligence quotient (IQ) and verbal comprehension index, while in girls, prenatal exposure had adverse effects on the full-scale IQ, the preceptive reasoning index, and the working memory index. Yet, the authors concluded that there was an adverse association between prenatal BPA exposure and cognitive development (Lin et al. 2017). Kim S et al*.* investigated the chemical exposure profile of women from the Children's Health and Environmental Chemicals in Korea (CHECK) cohort, in South Korea between 2011 and 2012, and subsequently associated it with their children's early neurodevelopmental performance at 13–24 months of age. Nonetheless, sex-dependent effects of BPA were observed, and it was found that there was an inverse association between urinary BPA levels and child mental developmental index (MDI) scores observed only among girls (Kim et al. 2018). In another epidemiological study, 506 Chinese pregnant women and their infants from the Laizhou Wan Birth Cohort (LWBC) from September 2010 to December 2013 were followed. Maternal BPA concentrations were adversely associated with children’s developmental quotient scores (DQ) at 12 months of age. Prenatal BPA concentrations were negatively associated with DQs in the adaptive domain among boys and girls, and DQs in the social domain only among girls (Pan et al. 2019). The effects of prenatal exposure to a mixture of EDCs, on neurodevelopment in school-aged children, were evaluated by the group of Tanner EM, and 718 mother–child pairs from the SELMA study were included. Among the phenols, BPA had the highest geometric means (GM) concentrations (1.53 ng/mL) and early prenatal exposure to EDCs among boys, was associated with lower intellectual functioning at age 7. Thus, it has been found that early prenatal exposure to a mixture of EDCs may be related to lower levels of cognitive functioning at age seven, particularly in boys (Tanner et al. 2020). On the other hand, from data from Wuhan Medical & Healthcare Center for Women and Children (WMHCWC), the relationships of repeated measurements of exposure to different bisphenols during pregnancy with child neurodevelopment in 456 mother–child pairs between 2014 and 2015 were evaluated. Second-trimester BPA concentrations were inversely associated with MDI scores (Jiang et al. 2020). Maternal urinary concentrations of chemical mixtures, including BPA, with IQ in children were also performed by Guo J et al*.* The data showed that prenatal exposure to BPA was associated with a decrease in the child's full intelligence quotient (FIQ) at age 7, thus affecting intellectual performance, especially in boys (Guo et al. 2020). Besides, Freire C et al*.* revealed in their study that BPA was associated in 4 to 5-year-old boys with poorer memory, verbal, and motor skills (Freire et al. 2020). Furthermore, Dualde P et al*.* implemented biomonitoring of biomarkers in children from Valencia, Spain, using urines from the BIOVAL program. The BIOVAL program is part of the Food Contaminants Exposure and Risk Assessment Program, which is focused on the biomonitoring of food contaminants in children from the Valencia Region. However, there are no observed negative effects after human exposure to BPA in children from this region (Dualde et al. 2021). On the other hand, Bornehag CG et al*.* saw the individual relationships between prenatal exposure to BPA, BPF and BPS and cognitive effects in 7-year-old children. Mothers were recruited into the SELMA study. As a result, BPA had a higher concentration of GM than BPF and BPS. However, BPA prenatal exposure had no significant association with effects on cognitive functions (Bornehag et al. 2021). Contrarily, Zheng J et al*.* also performed an epidemiologic study on mothers-child pairs and concluded that gestational exposure to low levels of BPA can cause damage to the developing brain at magnetic resonance imaging (MRI) resolution. Specifically, it was observed that maternal exposure to BPA can cause effects on the brain, in regions such as the postcentral gyrus, the opercular region of the inferior frontal gyrus and the superior occipital gyrus (Zheng et al. 2022). Otherwise, in the NHANES study, urinary levels of numerous phenols were assessed to see if they were related to the increased prevalence of antinuclear antibodies in adolescents and young adults. However, for BPA exposure, its levels showed no association with antinuclear antibodies (Parks et al. 2022).

#### Emotional neurobehavioral problems

Concerning BPA effects on neurobehaviour, several studies agreed that BPA can induce internalizing and externalizing problems, anxiety, and even depression, among other emotional problems. Yolton K et al*.* examined the association of prenatal exposure to BPA and phthalates, which included mother and infant pairs who were enrolled in the Health Outcomes and Measures of the Environment (HOME) study. Maternal urinary BPA metabolite concentrations at two distinct time points in pregnancy, at weeks 16 and 26 of gestation, were compared with scores on the NICU Neurobehavioral Network (NNNS), describing 13 dimensions of neurobehaviour, at 5 weeks of age in a cohort of 350 mother/infant pairs. However, prenatal BPA exposure was not associated with neurobehavioural outcomes at 5 weeks (Yolton et al. 2011). In the urine of Salinas Valley mothers during pregnancy in 1999–2000 and later in their children at age 5, the neurobehavioral effect of BPA was assessed. Higher prenatal BPA exposure is associated with depression and anxiety in boys at age 7. High urinary BPA concentration in children was also associated with internalizing problems (e.g. maladjusted emotional state), ADHD, and externalizing problems (e.g. inappropriate and uncontrolled behaviours such as aggressiveness, destruction of objects and hyperactivity) (Harley et al. 2013). In agreement, the group of Kundakovic examined DNA methylation of BDNF IV in cord blood samples from the Columbia Center for Children's Environmental Health (CCCEH) human cohort. As a result, high maternal exposure to BPA was shown to be associated with emotion regulation and increased aggressive behaviour in boys. Additionally, there was an alteration of BDNF expression and DNA methylation, which allows an association between the effects of BPA and several psychiatric disorders such as depression, schizophrenia, bipolar disorder, and ASD (Kundakovic et al. 2015). Moreover, Perez-Lobato R et al*.* analysed environmental BPA exposure of 300 children, belonging to the Environment and Childhood (INMA) Granada birth cohort, and assessed their child behaviour. Higher concentrations of BPA were associated with worse behavioural scores on all scales and BPA-exposed children had somatic complaints, thinking problems, and major social problems (Perez-Lobato et al. 2016). Pregnant women at 23–35 weeks of gestation between July 2002 and October 2005, from the Sapporo Toho Hospital in Hokkaido, Japan (Sapporo Cohort) were recruited by Minatoya M et al*.* No association between BPA level and either mental or psychomotor development at 6 and 18 months of age was found. However, it was found that at 42 months of age, the level of BPA at the umbilical cord was positively associated with increased scores of internalizing, externalizing, and developmental problems (Minatoya et al. 2017). Besides, Braun JM et al*.* used data from the Maternal-Infant Research on Environmental Chemicals (MIREC) study, which recruited 2,000 women in their first trimester of pregnancy from obstetric and prenatal clinics in Canada from 2008 to 2011. Prenatal urine BPA concentration was associated with some aspects of child behaviour, and some associations were stronger among boys (Braun et al. 2017b). The same group used the HOME study and followed 346 mother–child pairs (enrolled 2003–2006) from Cincinnati, OH, concluding that prenatal BPA concentrations were persistently associated with more externalizing behaviours (Braun et al. 2017c). On the other hand, Stacy SL et al*.* used data from the same study and showed that prenatal exposure to BPA was associated with externalizing behaviours in girls and at age 8 in boys (Stacy et al. 2017). In the Environment and Reproductive Health (EARTH) Study associations of BPA concentrations between parents and children were also analysed. Nonetheless, parental BPA concentrations were not associated with the Behavior-Assessment-System-for-Children-2 (BASC-2) index of behavioural symptoms, and internalizing or externalizing problems (Skarha et al. 2020). In contrast, the group of Li J explored the association between prenatal BPA exposure and childhood behavioural risks at ages 2 and 4 years, showing a positive association between maternal urinary concentrations of BPA during pregnancy and the risks of emotional problems, anxiety/depression problems, and internalizing problems in boys at ages 2 and 4 years. As in other studies, this association was more pronounced among boys (Li et al. 2020). Furthermore, this positive association were also observed with emotional symptoms, but not with cognitive and psychomotor development (Garí et al. 2021). Moreover, Liu J et al*.* investigated whether prenatal maternal exposure to BPA or BPS is associated with neurodevelopment in children at two years of age. Maternal selenium and child sex were thus found to be significant modifiers of the effect of BPA, which was negatively associated with socioemotional scores for boys but positive for girls. Still, higher exposure to bisphenols was associated with lower motor scores with lower maternal selenium levels by the Bayley Scales of Infant Development-III (Bayley-III) (Liu et al. 2021). England-Mason G et al*.* conducted a study in children between 2 and 4 years of age, recruited from the Alberta Pregnancy Outcomes and Nutrition (APrON) study. Child sex modified the associations between postnatal maternal BPA and changes in executive function, and thus higher concentrations of postnatal maternal BPA caused gradual problems in the domains of inhibitory self-control and emergent metacognition in girls (England-Mason et al. 2021). In another article, the same authors found an association between prenatal exposure to BPA and two of the high molecular weight phthalate-associated cytosine-phosphate-guanine sites in buccal epithelial cells, through maternal-child pairs recruited between 2009 and 2012 from the same data (England-Mason et al. 2022). The BPA exposure was also positively associated with adolescent thinking problems and somatic complaints in 130 boys from the INMA project (Mustieles et al. 2022).

#### Autism spectrum disorder (ASD)

Concerning the link between BPA exposure and ASD, Kondolot M et al*.* studied plasma levels of phthalates and BPA along with oxidant/antioxidant status in 51 autistic children admitted to Erciyes University Child Psychiatry Clinic in Kayseri between July 2011 and August 2012. 12 of these children were diagnosed with Pervasive Developmental Disorder-Not Otherwise Specified (PDD-NOS). As a result, plasma BPA levels of children with PDD-NOS were significantly higher than in autistic children and healthy children. Thus, was concluded that autistic children show increased oxidative stress due to increased exposure to BPA (Kondolot et al. 2016). From Danish participants from the Odense Child Cohort, Hansen JB et al*.* evaluated uterus exposure to BPA, with the aim of understanding if there is an association with ADHD and/or ASD. The data suggested an association in girls between BPA exposure and ASD score (Hansen et al. 2021). On the other hand, the group of Kim K quantified some types of phenols, such as BPA in 218 pregnant women from Markers of Autism Risk in Babies—Learning Early Signs (MARBLES). Although this study did not obtain results of cognitive effects (Kim et al. 2021). Four phenols were associated with ASD in the MARBLES study by Shin HM et al*.,* which concluded that diet was the major source of BPA exposure, and when comparing BPA urinary concentrations between MARBLES versus NHANES, the women that had a child with ASD, had lower concentrations of BPA urinary, as they were more aware/concerned about using BPA-rich utensils (Shin et al. 2021). Besides, Santos JX et al*.* also performed a study to identify 77 genes with high evidence of a link to ASD and then found that BPA interacts with 35 of these genes (Santos et al. 2022).

#### Attention-deficit/hyperactivity disorder (ADHD)

Regarding the association of the exposure of BPA with ADHD, Adesman A et al*.* examined the association between infant formula feeding and preschool ADHD in 2 comparable cohorts. For this study, the National Survey of Children's Health (NSCH) data from 2007 and 2011/2012, for children with ages 3 to 5 years, was used. ADHD was more common among formula-fed infants in 2007, but not in the 2011/2012 sample, where BPA exposure was markedly reduced, indicating that the reduced prevalence of ADHD among breastfed infants may not be due to the nutritional benefits of breast milk, but rather to early BPA exposure (Adesman et al. 2017). On the other hand, with data from the prospective Odense Child Cohort, the research group of Jensen TK investigated whether the concentration of BPA in maternal urine during pregnancy was associated with ADHD and language development in children aged 18 to 36 months. BPA was detected in 85.3% of maternal urine samples, and it was concluded that boys of mothers with high BPA concentration were 3.70 more likely to have a low language score (Jensen et al. 2019). Tsai CS et al*.* examined some EDCs, such as BPA, to understand whether they affect the gonadal hormones and whether this was related to ADHD susceptibility. Children with or without ADHD in the study were tested and it was concluded that the level of BPA was higher in boys with ADHD than in healthy boys (Tsai et al. 2020). Furthermore, Huang Z et al*.* analysed serum BPA concentrations and assessed behaviour and cognitive function in 3–4-year-old children from the China-Anhui Birth Cohort (C-ABCS). As a result, boys with the highest prenatal BPA concentrations showed an increase in conduct problems, while girls had behavioural and relationship problems. Therefore, high prenatal BPA concentrations have been shown to have a relationship with increased ADHD (Huang et al. 2022b). This association was also analysed in uterus exposure but no associations between BPA exposure and ADHD were observed (Hansen et al. 2021). On the other hand, Wang LJ et al*.* studied the differential levels between ADHD patients and healthy controls and no significant mediating effect of IGF-1 was found between BPA and ADHD (Wang et al. 2022b). Conversely, Kim JI et al*.* analysed the associations between BPA, BPF and BPS with ADHD symptoms at various periods of childhood, measuring BPA levels at 4, 6 and 8 years. ADHD symptoms were assessed using the ADHD Rating Scale IV (ARS). BPA was detected in about 97% of the participants, more than double that of other bisphenols. A doubling of BPA levels was associated with increased ARS scores by 4.7% at age 6 years, and there was therefore an association of BPA with attention-deficit/hyperactivity disorder above a threshold of 3.0 μg/g creatinine. However, BPA showed non-linear relationships with ADHD symptoms, but on the other hand, exposure to BPF and BPS was associated with greater ADHD symptoms at age 6 (Kim et al. 2022).

#### Stroke prevalence

Regarding the linking between BPA exposure and Stroke prevalence, still very weak and recently, the research group of Cai S associated for the first time Stroke with urinary BPA. This study was conducted with participants from the United States National Health and Nutrition Examination Surveys (NHANES) between 2003 and 2014. Therefore, a relationship was observed between urinary BPA and the increased prevalence of Stroke in the US population, and this was more evident in men, in 9139 participants, 324 people suffered from stroke (Cai et al. 2020).

In summary, the set of epidemiological studies presented suggests that BPA prenatal exposure seems to be related to the development of several neurological diseases in children like ADHD, ASD, depression, emotional problems (internalizing and externalizing problems), anxiety, cognitive disorders, and others (Table 4). Thus, several studies have always tried to relate the harmful effects of BPA on neurodevelopment in children, using remarkably diverse data. As for the adult studies, this consistency was not so clear, and of the four studies, only Cai et al. (2020) positively related urinary BPA with increased prevalence of Stroke and obtained worrisome results, but the remaining studies did not associate BPA with neurological diseases. Thus, we can infer that more prospective studies are needed to find the causal inference of BPA, and therefore caution is needed in their comparative analysis.

**Table 4:** Summaries of the disruptive effects of BPA in human epidemiological studies

Topic	Studied population	Gender	Age	Concentration (median)	Observed Effects	References
Neurodevelopmental problems	Pregnant women between February 2007 to February 2008 and their children in Heraklion, Crete, Greece	221 girls and 279 boys	4 to 4.48	1.9 µg/g creatinine (urinary)	Negative correlation with age and BPA exposure	Myridakis et al. (2016)
	198 mother–child pairs from a prospective cohort in Cincinnati, OH (HOME Study)	107 girls and 97 boys	8	2.0 μg/g creatinine median maternal urinary BPA	Maternal urinary BPA concentrations during pregnancy were not associated with VMWM performance	Braun et al. (2017a)
	Pregnant women living in Tarragona County (Spain) as part of the European “HEALS” project	45 mother–child pairs	–	0.72 µg/kg bw/day (0.28 to 1.42 µg/kg bw/day) from dietary intake	Foetus shows high sustained basal concentration of BPA due to low or no metabolic activity	Martínez et al. (2017)
	356 mother–child pairs from Taiwan Birth Panel Study	117 and 78 boys with 2 and 7 years, respectively; and 91 and 70 girls with 2 and 7 years, respectively	208 mother–child pairs with 2 years old children and 148 mother–child pairs with 7-year-old	3.3 ng/ml and 4.0 ng/ml for boys with 2 and 7 years, respectively 2.9 ng/ml and 1.2 ng/ml for girls with 2 and 7 years, respectively	In boys, significant associations were found in full-scale IQ and the verbal comprehension index In girls, prenatal BPA exposure had adverse effects on full-scale IQ, perceptual reasoning index, and working memory index Adverse association between prenatal BPA exposure and cognitive development in 7-year-olds but not in 2-year-olds	Lin et al. (2017)
	Pregnant women and their child’s from CHECK cohort, in South Korea between 2011 and 2012	71 girls and 69 boys	13 to 24 months (children)	4.0 μg/g creatinine (maternal urinary) 0.8 μg/L (breast milk)	Sex-dependent effects were observed for BPA Inverse association between urinary BPA levels and child MDI scores was observed only among girls	Kim et al. (2018)
	506 pregnant women and their child’s from LWBC from China between September 2010 and December 2013	188 boys and 180 girls in 1 year of age 152 boys and 144 girls in 2 years of age	1 and 2	1.05 μg/g creatinine (maternal urinary)	Maternal BPA concentrations were adversely associated with children DQs at 12 months of age Prenatal BPA concentrations were adversely associated with the adaptive domain DQs among boys and girls, and the social domain DQs only among girls	Pan et al. (2019)
	2300 pregnant women in the first trimester from prenatal clinics in Värmland county, Sweden, from November 2007 to March 2010	718 mother–child	7	1.53 ng/mL (maternal urinary)	Population-based pregnancy cohort, early prenatal exposure to a mixture of EDCs was related to lower levels of cognitive functioning at age seven, particularly among boys	Tanner et al. (2020)
	456 mother–child pairs from WMHCWC 2014–2015	245 boys and 211 girls	2	0.32 μg/L	Second-trimester BPA concentrations were inversely associated with MDI scores	Jiang et al. (2020)
	326 mother–child residing in Sheyang County of Jiangsu Province, China	347 children	7.4	2.78 μg/L (maternal urinary)	Negative associations between maternal BPA exposure and IQ scores in children aged 7 years Prenatal exposure to BPA were associated with decreased child’s FIQ	Guo et al. (2020)
	191 mother–child pairs from the INMA-Asturias, Gipuzkoa (Basque Country), Sabadell (Catalonia), and Valencia cohorts, between 2003 and 2008	105 boys and 86 girls	4 to 5	1.30 ng/g (placenta median BPA)	BPA was associated with boys having poorer verbal, memory, and motor skills	Freire et al. (2020)
	562 children from Valencia included in the BIOVAL program during 2016	282 boys and 280 girls	5 to 12	0.9 ng/mL	Internal exposure to BPA is of low concern for the Valencian children	Dualde et al. (2021)
	Women enrolled in the SELMA study from Värmland county, Sweden between November 2007 to March 2010	400 boys and 403 girls	7	1,55 ng/mL (GM urinary)	BPA had the highest GM concentrations No significant evidence of effects on cognitive functions	Bornehag et al. (2021)
	Pregnant women recruited between January 2009 and December 2012 form APrON	40 boys and 77 girls	8	2.25 ng/mL 1.42 ng/mL postnatal BPA	Maternal BPA exposure were associated with brain volume	Zheng et al. (2022)
	Participants of NHANES from 2003–2004 (*N* = 1023) and 2011–2012 (*N* = 918)	818 boys and 775 girls with negative ANA 57 boys and 135 girls with positive ANA	12 to 39	1.0 ng/ml (urinary)	BPA levels unrelated to antinuclear antibodies overall or by season	Parks et al. (2022)
Emotional neurobehavior problems	From March 2003 to February 2006, 389 healthy pregnant women’s residents in southwestern Ohio	350 mother/infant pairs enrolled in Health Outcomes and Measures of the Environment (HOME)	16-week pregnancy 26-week pregnancy	1.8 ng/mL (maternal urinary in 16-week pregnancy) 1.7 ng/mL (maternal urinary in 26-week pregnancy)	Higher BPA concentrations were associated with higher levels of maternal urine creatinine, serum cotinine, BMI, and with black race Maternal BPA concentrations were inversely associated with income and single marital status BPA during gestation was not associated with infant neurobehavior	Yolton et al. (2011)
	601 pregnant women and their children living in the Salinas Valley in 1999–2000	292 children’s	5	1.1 µg/L (maternal urinary) 2.5 µg/L (5-year-old children)	Higher prenatal BPA is associated with depression and anxiety in boys, at 7 years old Higher childhood BPA is related to internalizing problems and ADHD in boys and girls Higher childhood BPA is associated with externalizing problems in girls	Harley et al. (2013)
	Mothers recruited during pregnancy between 1998 and 2003 and children enrolled in the Columbia Center for Children’s Environmental Health (CCCEH) New York City (NYC) prospective cohort	87 boys and 111 girls	3 to 5	21 males and 22 females; BPA < 1 µg/L 19 males and 19 females; BPA > 4 µg/L	High maternal BPA exposure is associated with disturbed emotional regulation and increased aggressive behaviour in boys as well as with lower scores for anxiety/depression and reduced aggressive behaviour in girls BDNF expression and DNA methylation are associated the effects of BPA with several psychiatric disorders related to adversities in early life, including depression, schizophrenia, bipolar disorder, and autism	Kundakovic et al. (2015)
	300 children belonging to the INMA Granada birth cohort	300 boys	9 to 11	4.76 (2,77 to 9,03) μg/L	Higher BPA concentrations were associated with worse behavioural scores Children exposed to BPA had somatic complaints, thinking problems and major social problems	Perez-Lobato et al. (2016)
	Pregnant women at 23–35 weeks of gestation between July 2002 and October 2005 from the Sapporo Toho Hospital in Hokkaido, Japan (Sapporo Cohort)	127 boys and 158 girls	Tested at 6, 18, and 42 months of age	0.051 ng/ml (median level of cord blood BPA)	No association was found between BPA level and either mental or psychomotor development at 6 and 18 months of age BPA increased internalizing and externalizing problem scores of CBCL, in 42 months	Minatoya et al. (2017)
	2,000 women in the first trimester of pregnancy from Maternal–Infant Research on Environmental Chemicals (MIREC) study in Canada from 2008 to 2011	399 boys and 413 girls	2.8–4.2 years	0.8 (0.5 to 1.5) ng/ml	BPA was associated with more internalizing and somatising behaviours in boys, but not in girls	Braun et al. (2017b)
	346 mother–child pairs, from Cincinnati, OH, enrolled 2003–2006, from HOME study	188 girls and 158 boys	1 to 8	2.0 μg/g creatinine (maternal urinary)	Prenatal BPA concentrations were associated with more externalizing behaviours	Braun et al. (2017c)
	228 mothers-child pairs from HOME study between March 2003 and January 2006	127 girls and 101 boys	8	2.1 ng/mL prenatal BPA 222 ng/mL 8-Year BPA	Prenatal exposure to BPA was associated with more externalizing behaviours in girls and 8-year BPA was associated with more externalizing behaviours in boys	Stacy et al. (2017)
	220 parents and their children (n = 158) from EARTH study	127 mothers and 93 fathers 81 boys and 77 girls	2 to 9 (children)	1.2 μg/L in mothers and 1.7 μg/L in fathers	Parental BPA concentrations were not associated with BASC-2 behavioural symptoms index, internalizing or externalizing problems	Skarha et al. (2020)
	1225 pregnant women at 12–16 weeks of gestation residing in Shanghai, China	475 children at age 2 644 children at age 4	2 and 4	0.31 μg/L (urinary) at recruitment 0.12 μg/L (urinary) at age 4	BPA Maternal concentrations were associated with increased risks of Emotionally Reactive problem, Anxious/Depressed problem, and Internalizing problems in boys at 2 and 4 years of age	Li et al. (2020)
	Pregnant women (and their children) from REPRO_PL between 2007 and 2019	134 girls and 116 boys	1, 2 and 7	median 1.8 μg/l BPA (GM of 1.9 μg/l)	BPA exposure was positively associated with emotional symptoms, but not with cognitive and psychomotor development	Garí et al. (2021)
	Mothers and children from 2009 to 2012, with 1938 pregnant women residing in Calgary (Alberta, Canada)	394 mother–child pairs	2	1.7 μg BPA/g creatinine (urinary)	Maternal selenium and sex of the child were significant modifiers of BPA’s effect Maternal BPA was negatively associated with social emotional scores for boys only Higher exposure to bisphenols was associated with lower motor scores among children with lower levels of maternal selenium	Liu et al. (2021)
	Participants (n = 312) were recruited from APrON study from 2009 to 2012	158 boys and 154 girls	2 and 4	1.22 ng/mL (GM prenatal sample) and 0.93 ng/mL (GM postnatal sample)	BPA caused gradual problems in the domains of inhibitory self-control and emergent metacognition in girls	England-Mason et al. (2021)
	Maternal-child pairs recruited between 2009 and 2012 from APrON	76 boys and 76 girls	3 months	–	Prenatal BPA exposure was associated with two of the high molecular weight phthalate-associated cytosine-phosphate-guanine sites in buccal epithelial cells	England-Mason et al. (2022)
	Random sample of 668 mother-son pairs from INMA Project	130 boys	9 to 11	5.41 μg/g	Positive association with thinking problems and somatic complaints in adolescence BPA exposure associated with elevated BDNF DNA methylation	Mustieles et al. (2022)
ASD	Autistic children admitted to Erciyes University Child Psychiatry Clinic in Kayseri between July 2011 and August 2012	50 healthy children 51 autistic children	3.1 to 8.3	35 ng/ml BPA in healthy children and 37 ng/ml BPA in autistic children	Plasma BPA levels of children with PDD-NOS were significantly higher than control BPA exposure might be associated with PDD-NOS and oxidative stress	Kondolot et al. (2016)
	1739 mother–child pairs from prospective Odense Child Cohort between 2010 and 2012	570 boys and 509 girls	2 to 5	1,2 ng/mL (maternal urinary)	Significant dose–response relationship in girls between BPA exposure and ASD-score at 5 years of age	Hansen et al. (2021)
	218 California pregnant women participating in MARBLES during 2007–2014	218 women’s	20.5 to 49.2	1.0 ng/mL	BPA exposure decreased over the study period Non-pregnant women had higher BPA concentrations than pregnant women in NHANES study	Kim et al. (2021)
	173 pregnancies from MARBLES who have a child with ASD	164 women’s	91 women’s < 35 years and 82 ≥ 35 years	1.0 ng/mL	Diet was the major source of exposure	Shin et al. (2021)
	3,426 subjects (2,674 ASD cases and 752 unrelated ancestry-matched controls) from Autism Sequencing Consortium (ASC)	–	–	–	BPA exposure interacted with 35 genes among the 77 studied	Santos et al. (2022)
ADHD	NSCH children ages 3 to 5 years, in 2007 (weighted n = 9,644,405) and 2011/ 2012 (n = 9,732,865)	23 644 children	3 to 5	–	ADHD was more common among formula-fed infants in the 2007 but not the 2011/12 sample, where exposure to BPA was markedly reduced	Adesman et al. (2017)
	2874 pregnant women were recruited to the prospective OCC from 2010 to 2012	348 boys and 310 girls	3 and 4	1,2 ng/ml (maternal urinary)	Boys of mothers with high BPA were 3.70 more likely to have a low language score	Jensen et al. (2019)
	Children with ADHD recruited from Department of Child Psychiatry at Kaohsiung Chang Gung Children’s Hospital in Taiwan	98 boys and 32 girls with ADHD 42 boys and 26 girls without ADHD and any other psychiatric disorders	6 to 12	108.3 and 92.6 mg/dL (Urinary creatinine) for boys and girls with ADHD, respectively 104.1 and 107.7 mg/dL (Urinary creatinine) for healthy boys and girls, respectively	BPA level was higher in ADHD boys than control	Tsai et al. (2020)
	1739 mother–child pairs from prospective Odense Child Cohort between 2010 and 2012	570 boys and 509 girls	2 to 5	1,2 ng/mL (maternal urinary)	No associations between maternal BPA exposure and ADHD	Hansen et al. (2021)
	Patients with ADHD from the Department of Child Psychiatry at Chang Gung Memorial Hospital in Taiwan	110 boys and 34 girls with ADHD 46 boys and 24 girls	6 to 12	2.14 ng/mL for children with ADHD and 2.10 ng/mL for healthy children	BPA exposure was correlated with IGF-1	Wang et al. (2022b)
	Mother–child pairs from Congenital Anomaly Study from 2008–2010 619 children included in the analysis	325 boys and 294 girls	4, 6 and 8	3.9 μg/g creatinine (urinary) at age 4 2.36 μg/g creatinine (urinary) at age 6 1.96 μg/g creatinine (urinary) at age 8	Doubling in BPA levels was associated with increased ARS scores Association with Attention-deficit/hyperactivity disorder above a threshold of 3.0 μg/g creatinine BPA showed nonlinear relationships with ADHD symptoms	Kim et al. (2022)
	1,783 mother–child from the C-ABCS	1887 boys and 1386 girls	3 to 4	0.23 ng/mL (maternal serum BPA) 1.51 ng/mL (prenatal BPA)	Boys with the highest prenatal BPA concentrations showed an increase in conduct problems, while girls had behavioural and relationship problems High prenatal BPA concentrations have been shown to have a relationship with increased ADHD	Huang et al. (2022a, b)
Stroke	NHANES from 2003–2014 9139 adults aged ≥ 20 years	4467 men and 4672 women	≥ 20	≤ 1.00 to > 2.94 μg/g creatinine (urinary)	Association of BPA exposure with increased prevalence of stroke in U.S. population, more evident in males	Cai et al. (2020)

## Conclusions

In conclusion, BPA exposure is linked to an increasing prevalence of neurological disorders and diseases, based on the analysis of several BPA-induced neurological toxicity studies in animal models and humans. It’s now proved that BPA can have harmful effects on brain health, even when acting as an EDC at lower doses, especially during more vulnerable stages of development, such as pregnancy and the first years of a baby's life. In this analysis, it was seen that BPA exposure impairs various structural and molecular brain changes. In vitro, it was observed that BPA promotes oxidative stress, changing expression levels of several crucial genes and proteins. However, in vivo, was seen similar effects, adding to the destructive effects on neurotransmitters, excitotoxicity and neuroinflammation, and damaged blood–brain barrier function, leading to deteriorated cognitive functions and cholinergic system, impaired spatial learning and memory capacity, neurodegenerative diseases, and neuropsychological disorders. On the other hand, human in vitro studies showed effects such as neuronal damage, apoptosis effects, disruption of intracellular Ca^2+^ homeostasis, a significant increase in ROS, promoted apoptosis and LDH release, decreased axon length, microglial DNA damage, astrogliosis, BCRP inhibition, and significantly reduced myelination. Finally, epidemiological studies have demonstrated the harmful effects of BPA on neurodevelopment in children leading to the development of considerable neurological diseases like ADHD, ASD, depression, emotional problems, anxiety, and cognitive disorders. However, this type of study also reveals a positively related urinary BPA with increased prevalence of Stroke, which are worrying results.

Therefore, it becomes obvious that human daily exposure is not safe and that the issues caused by the widespread usage of this EDC are still present. The various experimental methods used, as well as adjustments to dose amounts, exposure times, or even the experimental model employed, might be used to support the contentious research. In this regard, more studies are needed to further clarify the mechanism of BPA toxicity in the neurological system. Furthermore, the fact that humans are frequently exposed to numerous endocrine disruptors, including bisphenols, highlights the necessity of researching co-exposure patterns and/or the “exposome” to best address the negative effects of this EDC on neurological health while always considering multiple exposures. A crucial stage in the progress of research on these substances is the creation of new BPA analogues as well as the examination of the safety of those analogues already created today. There should be no health concerns associated with environmental or human exposure to these substances, especially for the most susceptible populations like children and pregnant women. It's crucial to use public health strategies to determine the best ways to reduce BPA exposure. This includes promoting the use of glass containers for food storage and reducing the use of canned goods, plastic containers, water supply pipes and many more products that contain BPA. This will improve people's quality of life and reduce the risk of BPA-induced neurological toxicity.

## Data Availability

Data sharing not applicable to this article as no datasets were generated or analysed during the current study.

## Abbreviations

5-HT: Serotonin
ACh: Acetylcholine
AChE: Acetilcolinesterase
ADHD: Attention-deficit/hyperactivity disorder
AR: Androgen receptors
ASD: Autism spectrum disorder
Aβ: Amyloid-β
BADGE: Bisphenol A diglycidyl ether
BCRP: Breast cancer resistance protein
BDNF: Brain-derived neurotrophic factor
BPA: Bisphenol A
BPAF: Bisphenol AF
BPB: Bisphenol B
BPF: Bisphenol F
BPS: Bisphenol S
Ca^2+^: Calcium
CAT: Catalase
DA: Dopamine
DRP-1: Dynamin-related protein 1
EDC: Endocrine-disrupting compound
EGCG: Epigallocatechin-3-gallate
ER: Estrogen receptors
GABA: Gamma-aminobutyric acid
GFAP: Glial fibrillary acidic protein
GnRH: Gonadotropin-releasing hormone
GSH: Glutathione
GST: Glutathione-*S*-transferase
HDAC2: Histone deacetylase
hESC: Human embryonic stem cells
hiPSC: Human induced pluripotent stem cell
HOME: Health Outcomes and Measures of the Environment
IGF-1: Insulin-like growth factor 1
LDH: Lactate dehydrogenase
lncRNA: Long non-coding RNAs
MARBLES: Markers of Autism Risk in Babies-Learning Early Signs
MBP: 4-Metil-2,4-bis(4-hidroxifenil)pent-1-eno
MCAo: Middle cerebral artery occlusion
MCAO/R: Middle cerebral artery occlusion and reperfusion
MCP1: Monocyte chemoattractant protein-1
MDA: Malondialdehyde
MetS: Metabolic syndrome
miRNA: MicroRNAs
MMP: Mitochondrial membrane potential
mRNA: Messenger RNA
NHANES: United States National Health and Nutrition Examination Surveys
NO: Nitric oxide
NOS: Nitric oxide synthases
NPPs: Nano-sized plastic particles
NSC: Neural stem cells
OGA: O-GlcNAcase protein
OXPHOs: Oxidative phosphorylation
PDI: Protein disulfide isomerase
PFC: Prefrontal cortex
PPAR-γ: Peroxisome proliferator-activated receptor gamma
PSD-95: Postsynaptic density protein-95
p-tau: Phosphorylated tau protein
PUMA: P53 upregulated modulator of apoptosis
ROS: Reactive oxygen species
SELMA: Swedish Environmental Longitudinal, Mother and child, Asthma and allergy
CNS: Central nervous system
SOD: Superoxide dismutase
Syn2α: Anti-synapsin 2α
SYP: Synaptophysin
γ-IR: γ-Radiation
